# Neuroinflammation, myelin and behavior: Temporal patterns following mild traumatic brain injury in mice

**DOI:** 10.1371/journal.pone.0184811

**Published:** 2017-09-14

**Authors:** Toufik Taib, Claire Leconte, Juliette Van Steenwinckel, Angelo H. Cho, Bruno Palmier, Egle Torsello, Rene Lai Kuen, Somfieme Onyeomah, Karine Ecomard, Chiara Benedetto, Bérard Coqueran, Anne-Catherine Novak, Edwige Deou, Michel Plotkine, Pierre Gressens, Catherine Marchand-Leroux, Valérie C. Besson

**Affiliations:** 1 EA4475 – Pharmacologie de la Circulation Cérébrale, Faculté de Pharmacie de Paris, Université Paris Descartes, Sorbonne Paris Cité, Paris, France; 2 U1141 PROTECT, INSERM, Université Paris Diderot, Sorbonne Paris Cité, Paris, France; 3 Cellular and Molecular Imaging Platform, CRP2, UMS 3612 CNRS, US25 INSERM, Faculté de Pharmacie de Paris, Université Paris Descartes, Sorbonne Paris Cité, Paris, France; University of Florida, UNITED STATES

## Abstract

Traumatic brain injury (TBI) results in white matter injury (WMI) that is associated with neurological deficits. Neuroinflammation originating from microglial activation may participate in WMI and associated disorders. To date, there is little information on the time courses of these events after mild TBI. Therefore we investigated (i) neuroinflammation, (ii) WMI and (iii) behavioral disorders between 6 hours and 3 months after mild TBI. For that purpose, we used experimental mild TBI in mice induced by a controlled cortical impact. (i) For neuroinflammation, IL-1b protein as well as microglial phenotypes, by gene expression for 12 microglial activation markers on isolated CD11b^+^ cells from brains, were studied after TBI. IL-1b protein was increased at 6 hours and 1 day. TBI induced a mixed population of microglial phenotypes with both pro-inflammatory, anti-inflammatory and immunomodulatory markers from 6 hours to 3 days post-injury. At 7 days, microglial activation was completely resolved. (ii) Three myelin proteins were assessed after TBI on ipsi- and contralateral *corpus callosum*, as this structure is enriched in white matter. TBI led to an increase in 2',3'-cyclic-nucleotide 3'-phosphodiesterase, a marker of immature and mature oligodendrocyte, at 2 days post-injury; a bilateral demyelination, evaluated by myelin basic protein, from 7 days to 3 months post-injury; and an increase in myelin oligodendrocyte glycoprotein at 6 hours and 3 days post-injury. Transmission electron microscopy study revealed various myelin sheath abnormalities within the *corpus callosum* at 3 months post-TBI. (iii) TBI led to sensorimotor deficits at 3 days post-TBI, and late cognitive flexibility disorder evidenced by the reversal learning task of the Barnes maze 3 months after injury. These data give an overall invaluable overview of time course of neuroinflammation that could be involved in demyelination and late cognitive disorder over a time-scale of 3 months in a model of mild TBI. This model could help to validate a pharmacological strategy to prevent post-traumatic WMI and behavioral disorders following mild TBI.

## Introduction

Traumatic brain injury (TBI) is a leading cause of mortality and disability that mainly affects young adults in industrialized countries and that imposes a substantial social and economic burden on the community [1,2]. As primary injury occurs immediately after trauma, prevention is the only possibility to limit this type of injury. However, the latter leads to secondary injury such as white matter injury (WMI) and neuroinflammation [3]. These events may develop from hours to days, weeks, months or even years following the impact, providing a window of opportunity for therapeutic intervention.

WMI is commonly observed in surviving TBI patients and is associated with severe neurological deficits and impaired quality of life [4,5]. White matter disruption has been described from the early phases to years after injury in both mild and severe TBI patients [6–9]. WMI is characterized by both axonal damage and myelin pathology. Axonal damage includes traumatic and diffuse axonal injury, with axonal loss caused by Wallerian degeneration and/or cavitation injury [10,11]. Myelin pathology can result from either loss of myelin due to loss of axons, and/or from secondary damage that cause oligodendrocyte loss with subsequent demyelination of viable/intact axons [12]. Oligodendrocyte death has been reported in animal model of TBI [13–15] as well as in humans [16]. Demyelination was recently evidenced in mice 12 months after mild TBI [17,18].

Neuroinflammation is observed in both acute and chronic stages after moderate/severe TBI in human [9] and in animal models of TBI [19–21]. In post-mortem brains, Johnson and colleagues [9] have showed neuroinflammation, characterized by microglial activation, that persists many years after TBI, and is associated with WMI. Similar findings have been described in models of TBI [22,23], suggesting that neuroinflammation might participate in WMI. While microglia activation has been considered detrimental, it is now recognized that it may also promote protective and regenerative effects. In fact, two phenotypes of activated microglia called classical and alternative activation have been described. To date, the classical activation is associated with pro-inflammatory and detrimental effects, whereas the alternative activation of microglia mediates anti-inflammatory, regenerative thus beneficial effects [24–28]. In animal TBI models, mixed populations of microglia with both phenotypes were observed [29–32].

The controlled cortical impact (CCI) model is the one of the most commonly used animal models of pre-clinical TBI. Its main advantages are the possibility to control injury parameters (velocity, depth, time) to produce a wide range of TBI severities, and its ability to reproduce many of human TBI aspects [33]. Both neuroinflammation and WMI were described in CCI model in several studies. However, all data were obtained using moderate to severe TBI. As to date, there is no data on mild TBI, we investigated the time courses of neuroinflammation, mainly microglial phenotypes, WMI, and behavior at selected time points ranging from 6 hours to 3 months after a mild TBI induced by CCI in mice.

## Materials and methods

### Animals

All care and experiments were in accordance with the ethical approvals stipulated by the Animal Ethics Committee of Paris Descartes University, the French regulations and the European Union Council Directive of September 22, 2010 (2010/63/EEC) on the protection of animals for experimental use (APAFiS#4765; APAFiS#6633) and conformed to the Guide for the Care and Use of Laboratory Animals published by the U.S. National Institutes of Health (publication 85–23, revised 1996). Male Swiss mice were supplied by Janvier Labs (Le Genet St Isle, France). Mice were randomly assigned to groups as follows: unoperated, sham-operated and TBI.

### Controlled cortical impact-induced brain injury

**Fig 1:**
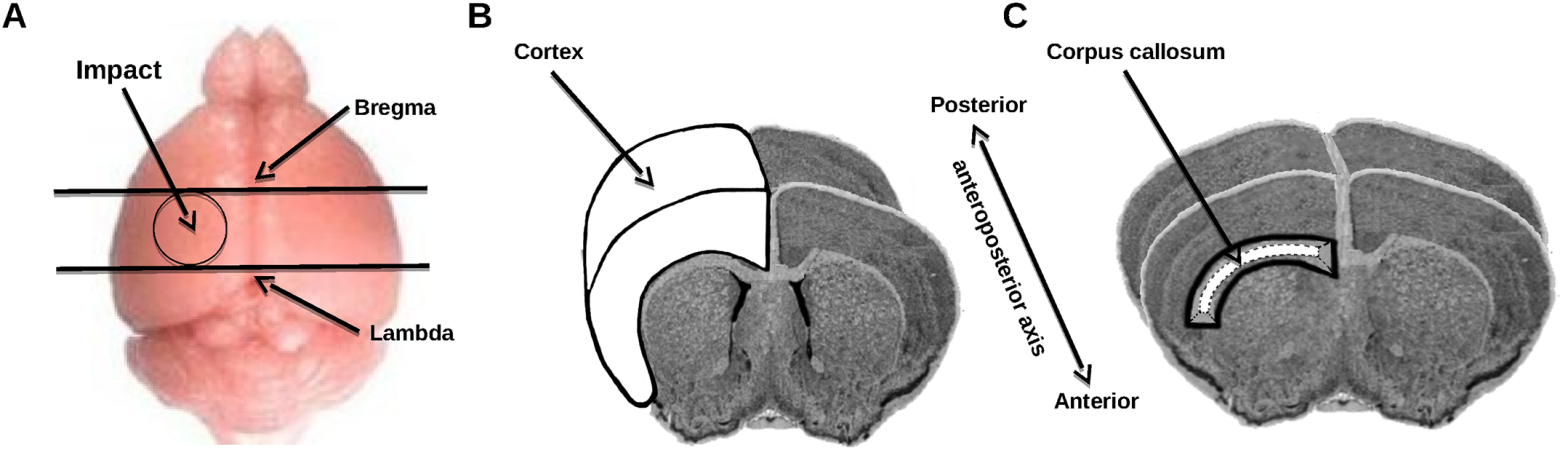
Schematic representations of area of impact and brain samples. (A) Area of cortical impact, (B) brain sample (ipsi- and contralateral *cortex*) used for IL-1b and (C) brain sample (ipsi- and contralateral *corpus callosum*) used for myelin proteins study.

The controlled cortical impact (CCI) model was performed as previously described [34]. Mice, weighing 28 to 30g, were anesthetized with isoflurane and placed in a stereotaxic frame. Body temperature was monitored throughout surgery by a rectal probe and maintained at 37.0±0.5°C with a homeothermic blanket control unit. A 4-mm craniotomy was performed onto the left temporo-parietal cortex centered between the bregma and lambda, taking care to leave the *dura mater* intact (Fig 1A). Injury was delivered using a 3-mm diameter impactor by a pneumatically controlled device (TBI 0310 Impactor, Precision System Instruments) using these parameters: diameter 3mm, velocity 3.5m/s, depth of cortical deformation 1.0mm and dwell time 50ms [10]. Following the injury, the skullcap was replaced by applying bone wax and the skin sutured. Sham-operated mice underwent the same surgery without impact. To recover from anesthesia and prevent post-surgery hypothermia, animals were placed in an incubator set at 30°C for one hour. Mice were subsequently returned to their home cage and housed under temperature- and light-controlled conditions with access to food and water *ad libitum*.

### Experiments

**Fig 2:**
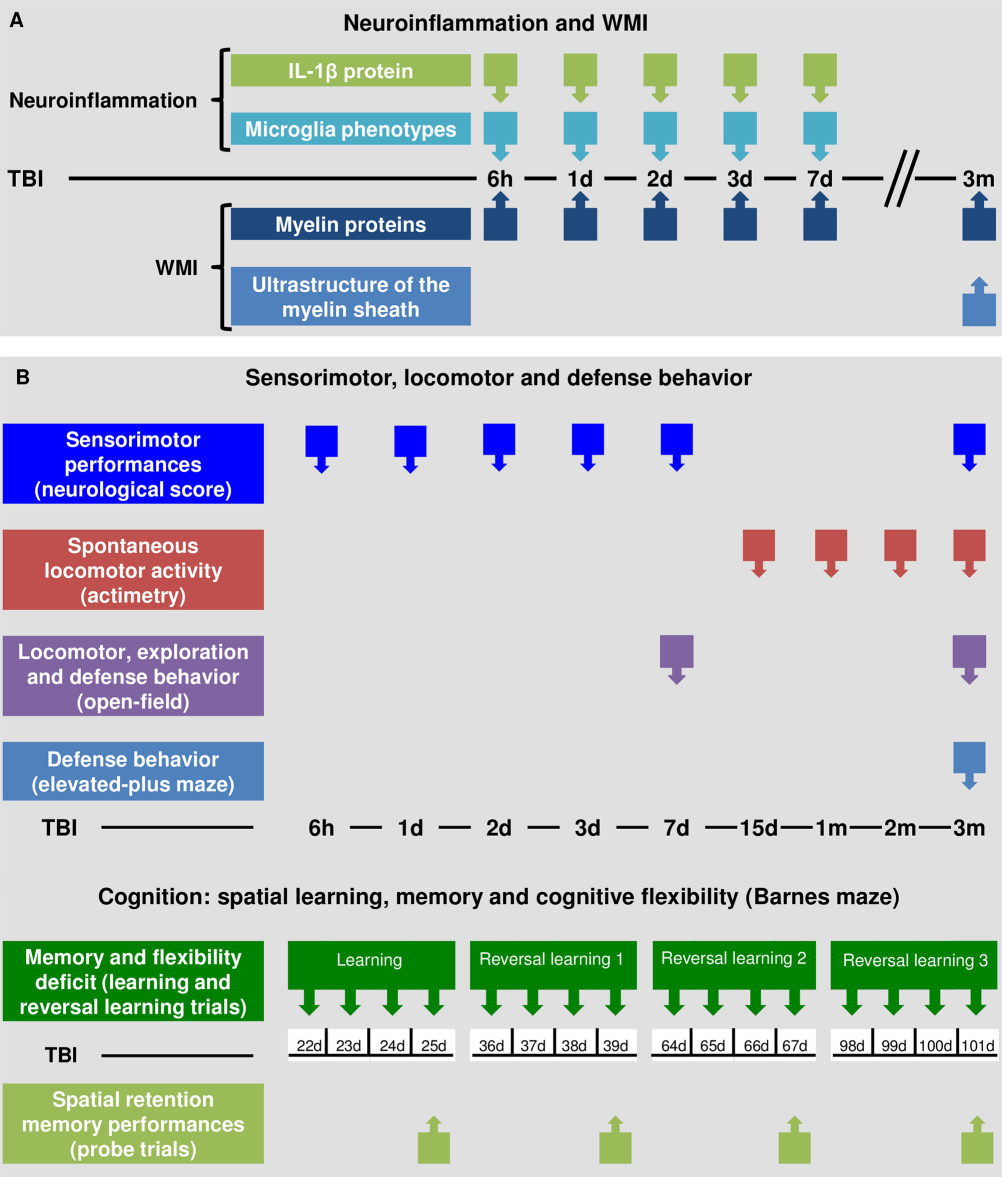
Outline of the experimental studies and time points studied. Schedule of (A) neuroinflammation and WMI studies, and (B) behavioral studies.

Three studies were performed according to the Fig 2.

#### Study 1: Time course of neuroinflammation evaluated by IL-1b and microglial phenotypes

IL-1b protein was measured in the contusion (cortical area) at 6 hours, 1, 2, 3 and 7 days in unoperated (n = 12), sham-operated (n = 12) and TBI (n = 6-7/time-point) mice. Microglial phenotypes (pro-, anti-inflammatory and immunoregulatory) were evaluated at 6h, 1, 2, 3, and 7 days in sham-operated (n = 14/time-point) and TBI (n = 12-16/time-point) mice.

IL-1b assay: 3-mm thick ipsi- and contralateral cortices (Fig 1B) were homogenized in ice-cold buffer, then centrifuged as previously described [35,36]. IL-1b levels were measured using a commercialized ELISA kit (RetD systems Europe Ltd, Abingdon, United Kingdom) according to the manufacturer’s instructions.

CD 11b-positive cell selection: anesthetised mice were intracardially perfused with 0.9% NaCl and brains were rapidly collected. After removing the cerebellum and olfactory bulbs, brains were dissociated using the Adult Brain Dissociation Kit and the gentleMACS Octo Dissociator with Heaters (Miltenyi Biotec, Germany). The resulting brain homogenates from two brains were pooled and cleaned of their debris using the debris removal solution of the kit according to the manufacturer’s instructions. CD11b^+^ cells were enriched using the anti-CD11b (microglia) MicroBeads (Miltenyi Biotec, Germany) and multiMACS Cell24 separator (Miltenyi Biotec, Germany). After elution, the CD11b^+^ isolated cells were centrifuged for 10 min at 300g and the pellets were conserved at -80°C.

Real-time qPCR analysis: total RNA was extracted with the RNeasy micro kit according to the manufacturer’s instructions (Qiagen, France). RNA quality and concentration were assessed by spectrophotometry using the NanodropTM apparatus (Thermofisher Scientific, MA, USA). A reverse transcription using the iScriptTM cDNA synthesis kit (Bio-Rad, France) was realized on 300 ng of total RNA. RT-qPCR was performed in triplicate for each sample using SYBR Green Super-mix (Bio-Rad) for 40 cycles with a 2-step program (5 s of denaturation at 95°C and 10 s of annealing at 60°C). Amplification specificity was assessed with a melting curve analysis. Primers were designed using Primer3 plus software, and sequences and their NCBI references are given in Table 1. Specific mRNA levels were calculated after normalization of the results for each sample with those for *Rpl13a* mRNA, used as a reference gene. The data are presented as relative mRNA units with respect to control group (expressed as fold over control value).

**Table 1:** Primer sequences.

Gene	Target protein and abbreviation	Forward	Reverse
*Rpl13a*	Ribosomal protein L13 a	ACA GCC ACT CTG GAG GAG AA	GAG TCC GTT GGT CTT GAG GA
*Cd32*	Cluster of differentiation 32	CTG GAA GAA GCT GCC AAA AC	CCA ATG CCA AGG GAG ACT AA
*Cd86*	Cluster of differentiation 86	GAG CGG GAT AGT AAC GCT GA	GGC TCT CAC TGC CTT CAC TC
*Ptgs2*	Prostaglandin-Endoperoxide Synthase 2 (= Cox2)	TCA TTC ACC AGA CAG ATT GCT	AAG CGT TTG CGG TAC TCA TT
*CD206*	Cluster of differentiation 206	CTT CGG GCC TTT GGA ATA AT	TAG AAG AGC CCT TGG GTT GA
*Lgals3*	Lectin Galactoside-Binding Soluble 3	GAT CAC AAT CAT GGG CAC AG	ATT GAA GCG GGG GTT AAA GT
*Igf1*	Insulin like growth factor 1	TGG ATG CTC TTC AGT TCG TG	GCA ACA CTC ATC CAC AAT GC
*Il1rn*	Interleukin 1 receptor antagonist	TTG TGC CAA GTC TGG AGA TG	TTC TCA GAG CGG ATG AAG GT
*Il4ra*	Interleukin 4 receptor alpha	GGA TAA GCA GAC CCG AAG C	ACT CTG GAG AGA CTT GGT TGG
*Socs3*	Suppressor of cytokines 3	CGT TGA CAG TCT TCC GAC AA	TAT TCT GGG GGC GAG AAG AT
*Tnfa*	Tumor necrosis factor alpha	GCC TCT TCT CAT TCC TGC TT	AGG GTC TGG GCC ATA GAA CT
*Il1b*	Interleukin 1b	GGG CCT CAA AGG AAA GAA TC	TCT TCT TTG GGT ATT GCT TGG
*Il10*	Interleukin 10	CTC CCC TGT GAA AAT AAG AGC	GCC TTG TAG ACA CCT TGG TC

Nomenclature of microglial phenotype: we have adopted nomenclature consistent with the previous work in primary microglia [24]. We distinguished 3 types of phenotypes according to the mRNA expression levels of markers listed in brackets: pro-inflammatory (*Il1b*, *Tnfa*, *Ptgs2*, *Cd32 and Cd86*), anti-inflammatory (*Lgals3*, *Igf1*, *Cd206 and Il10*) and immunoregulatory (*Il1rn*, *Il4ra and Socs3*).

#### Study 2: Time course of white matter injury evaluated by myelin proteins and ultrastructure of the myelin sheath

To investigate white matter injury, expression of 3 myelin proteins and ultrastructural modifications of fibers were investigated in the *corpus callosum*, a cerebral area enriched with white matter.

To evaluate myelin proteins, sham-operated (n = 12) and TBI (n = 5-6/time-point) mice were anesthetised (150 mg/kg sodium pentobarbital, i.p.) and sacrificed by decapitation at 6 hours, 1, 2, 3 and 7 days, and 3 months post-injury. Three-mm thick ipsi- and contralateral *corpus callosum* (Fig 1C) were rapidly dissected for western blot of myelin basic protein (MBP), 2',3'-cyclic-nucleotide 3'-phosphodiesterase (CNPase), myelin oligodendrocyte glycoprotein (MOG) and actin in *corpus callosum*. MBP was used as a marker of myelination [37]. CNPase as a marker of immature and mature myelinating OL [38] and MOG as a minor myelin protein expressed by OL in the later stages of myelination [39].

To observe ultrastructural modifications of the myelin sheath in *corpus callosum*, sham-operated (n = 1) and TBI (n = 1) mice were anesthetised (150 mg/kg sodium pentobarbital, i.p.) and transcardially perfused with 0.9% saline followed by a fixative solution (2% paraformaldehyde, 2.5% glutaraldehyde, 0.1 M C_2_H_6_AsNaO_2_, 3mM CaCl_2_ buffer, pH = 7.4) at 3 months post-injury. Brains were removed and post-fixed at 4°C overnight in the fixative solution for transmission electron microscopy (TEM) analysis of the *corpus callosum*.

Western-blot of myelin basic protein, 2',3'-cyclic-nucleotide 3'-phosphodiesterase, myelin oligodendrocyte glycoprotein and actin: 3-mm thick ipsi- and contralateral *corpus callosum* were homogenized and centrifuged. After electrophoresis, proteins were transferred to a polyvinylidene difluoride membrane. Membranes were incubated with primary antibody [rabbit anti-MBP antibody and rabbit anti-actin antibody (for membranes used for western-blot of MBP); rabbit anti-MOG antibody and rabbit anti-actin antibody (for membranes used for western-blot of MOG)] in blocking solution. Membranes were then washed and incubated with the appropriate secondary (donkey anti-rabbit-FITC IgY secondary polyclonal antibody). For western-blot of CNPase, the membranes used for western-blot of MBP were incubated in a dehybridization buffer and then incubated with chicken anti-CNPase primary antibody in blocking solution. Membranes were then washed and incubated with the appropriate secondary antibody (donkey anti-chicken IgY secondary antibody FITC conjugate). The signal was then amplified through the incubation of a rabbit anti-FITC-Alkaline Phosphatase. Finally, enhanced chemi fluorescent (ECF) reactive was added to the membrane. The alkaline phosphatase dephosphorylates the ECF substrate resulting in a fluorescent product. Membranes were scanned by immunofluorescence using a Storm^™^ 860 (GE Healthcare Life Sciences) and ImageQuant^™^ software (GE Healthcare Life Sciences) for quantification. Normalization was performed by dividing the MBP signal value by the corresponding actin signal value. MBP, CNPase and MOG expression in the controlateral *corpus callosum* of sham-operated was considered as 100%. Then, MBP, CNPase and MOG expression in the ipsilateral *corpus callosum* of sham-operated and TBI were calculated relative to 100%.

Transmission electron microscopy: following the post-fixation, brains were hemisected along the midline. Ipsilateral (left side) hemispheres were cut on a vibratome (Leica VT 1200S) into 300-μm sagittal sections. The *corpus callosum* was carefully isolated, and divided into three blocks corresponding to the three callosal regions (genu, body, and splenium). Briefly, tissue blocks were rinsed in 0.1 M C_2_H_6_AsNaO_2_, then fixed in 1% OsO_4_ for 1 h at 4°C. After a series of washing in C_2_H_6_AsNaO_2_ and distilled water, tissue blocks were stained with 1% uranyl acetate for 2 h at room temperature. Graded concentrations of ethanol, followed by propylene oxide, were used to dehydrate the tissue. The dehydrated tissue blocks were incubated in a 50/50 mixture of epon/propylene oxide for 2 h, followed by 100% epon overnight and then twice 100% epon for 3 h. The tissue blocks were placed in an embedding mold using 100% epon, which was polymerized through incubation at 37°C for 1 day followed by an incubation at 60°C for 2 days. Semi-thin sections, 0.5 μm thick, were generated for each block, using an ultramicrotome (ULTRACUT, Reichert-Jung). These sections were heat dried on glass slides and stained with methylene blue for evaluation of region of interest (ROI) within *corpus callosum* by light microscopy. For electron microscopy, the tissue around the ROI was trimmed. Ultrathin sections (80 nm) were then cut, using an ultramicrotome (ULTRACUT, Reichert-Jung). The ultrathin sections were mounted on copper grids. Grids were then stained with lead citrate. Morphological alterations of the myelin sheath were explored in a selected ROI of each callosal regions (genu, body, and splenium) in unoperated, sham-operated and TBI mice. Ultrastructural myelin abnormalities were observed using the TEM (Japan Electron Microscope-100S) in the Cellular and Molecular Imaging facility, INSERM UMS 025 –CNRS UMS 3612, Faculty of Pharmacy of Paris, Paris Descartes University.

Ten fields per grid were selected per condition (two grids per condition; total of 20 fields per condition). Five random images were collected within each field (100 images, area = 751 μm^2^) at x4 000 magnification. Myelinated axons, that were fully in the image, were counted. Then the percentages of axons showing either fragmentation or decompaction of the myelin sheath, or separation of myelin from the axon, or minimum two of these abnormalities, were calculated for sham-operated and TBI.

#### Study 3: Time course of sensorimotor, locomotor, defense behavior and cognitive performances

Experiment 1: evaluation of sensorimotor performances (neurological score) locomotor, exploration and defense behavior (open-field) on unoperated (n = 6), sham-operated (n = 5) and TBI (n = 7) mice from 6 hours to 7 days.

Sensorimotor performances were evaluated at 6 hours, 1, 2, 3 and 7 days post-injury, using the neurological score, as it revealed sensorimotor deficits after focal ischemia in mice [40].

Unconditioned exploration and defense behavior were assessed at 7 days post-injury on these animals, using the open-field test [41].

Experiment 2: As unconditioned defense behavior is based on exploration of a novel environment, evaluation of sensorimotor performances (neurological score), spontaneous locomotor activity (actimetry, [42]), exploration and defense behavior (open-field and elevated-plus maze), and spatial learning, memory and cognitive flexibility (Barnes maze, [43]) later than 7 days post-injury were performed on other unoperated (n = 11), sham-operated (n = 12) and TBI (n = 9) mice.

Actimetry test was performed at 15 days, 1, 2 and 3 months after the surgery.

The neurological score, the open-field and the elevated-plus maze tests were performed at 3 months.

Barnes Maze test was performed at 15 days (learning: day 22, 23, 24 and 25), 1 month (reversal learning 1: day 36, 37, 38 and 39), 2 months (reversal learning 2: day 64, 65, 66 and 67) and 3 months (reversal learning 3: day 98, 99, 100 and 101) after the surgery.

Neurological score: neurological evaluation was performed, in a blind manner, by attributing a grade according to the severity of the deficit evaluated for several items: no circling toward the paretic side, resistance to left and right push, and tactile stimulation of the two ears; other items were evaluated when the mouse was raised by the tail: no flexion of the body, the forelimbs and hindlimbs. The maximal neurological score was 20 (each of the 10 items are scored on 2 points), attesting the absence of deficit.

Actimetry: the horizontal (locomotion) and vertical (rearings) activities were individually assessed in transparent activity cages (20x10x12cm) with automatic monitoring of photocell beam breaks (Imetronic, Bordeaux, France). Actimetry test was performed at days 14, 29 (1 month), 56 (2 months), 91 (3 months), with recording every 10 min over 1 h, in order to evaluate the effect of TBI or surgery on spontaneous locomotor activity, as modified locomotor activity could lead to biased results from other behavioral tests requiring locomotion.

Open-field: the open-field test was performed using a square white open-field apparatus (40x40x45cm) made of plastic permeable to infrared light. Distance traveled (evaluating locomotor activity), time spent in the center of the open-field (evaluating defense behavior), were recorded by a videotrack system (Viewpoint) during the 9 min test. A blinded investigator counted the number of rearings (evaluating exploration behavior).

Elevated-plus maze test: defense behavior was evaluated using the elevated-plus maze that relies on the animals’ preference for dark enclosed arms over bright open arms. This task assesses the willingness of the mouse to explore the open arms of the maze that are fully exposed and at an elevated height. Time spent in the open arm is decreased in mice that exhibit anxiety-like behavior. The maze consisted of a Plexiglas plus-shaped platform elevated 50 cm from the floor with 4 arms intersecting at a 90° angle, creating 4 individual arms each 37 cm long and 6 cm wide. The 2 closed arms were shielded by 14 cm-high side and end walls, whereas the 2 open arms had no walls. Arms are connected each other’s by a central platform (6x6cm).

The mouse was placed on the central platform, facing one of the open arms. The mouse was allowed to explore the maze for a 9-min period (540s) while the number of entries and the time spent in each of the arms were recorded by a videotrack system (Viewpoint) every 3 min. As advised by File and coll. [44] data were presented as % time spent in the open arms = [(time spent in open arms entries/(total time: 540s) x 100].

Barnes maze test: the Barnes maze test was performed at 15 days (day 22 to 25), 1 month (from day 36 to 39), 2 months (from day 64 to 67), and 3 months (from day 98 to 101). The maze is a wet, white and circular platform (80cm diameter), brightly illuminated (400lux), raised 50cm above the floor, with 18 holes (5cm) equally spaced around the perimeter. A white hidden escape box (8x5x5cm), representing the target, was located under one of the holes.

Prior to the test, each mouse was subjected to a habituation trial where the mouse was directly put in the escape box for 30 s.

Learning: the mouse was placed in the center of the circular maze and was allowed to explore the platform and holes for 3 min maximum. The distance traveled was recorded using a videotrack system (Viewpoint). Latency to reach the escape box, and number of errors (number of empty holes visited) were manually noted. When the mouse found and entered the escape box, the videotrack recording was stopped, and the mouse waited the experimenter for 10 s before returning to its home cage. If the mouse did not enter spontaneously, it is gently put toward the escape box, before returning to its home cage. On the first day of training, mice underwent 2 trials after the habituation trial; thereafter, 3 trials were given per day, with a 2 h intertrial interval.

Probe trial: on the 4^th^ day of learning (or reversal learning), the probe trial, with the escape box removed and lasting 1 min, is used to assess spatial memory performance. Time spent and distance traveled in each sextant (defined by one of the 6 parts of the maze, that includes 3 holes) were recorded. The target zone is defined as the part, which contains the target hole and two adjacent holes.

Reversal learning: mice underwent 3 trials per day, with a 2 h intertrial interval during four days in order to learn a new location of the escape box. On the 4^th^ day, a probe trial was performed.

### Statistical analysis

Data were expressed as mean ± SEM of *n* observations, where *n* represents the number of animals used. All statistical graphs and analyses were created with GraphPad Prism 5.0 (GraphPad Software, San Diego, CA).

As normality test failed for IL-1b, MBP, CNPase, MOG expression, non-parametric Kruskal-Wallis analysis with subsequent comparison by Mann-Whitney U test were performed. For microglia phenotypes, differences were analyzed by 2-way ANOVA with subsequent group comparisons by Dunnett test with Bonferroni correction.

For neurological score, actimetry and Barnes maze test, differences were analyzed by 2-way ANOVA for repeated measures, followed by a Dunnett test with Bonferroni correction. For the Barnes maze probe trial, univariate t test was performed to compare the % time spent in the target sextant to the theoretical value 16.67% (i.e. when the mouse spent equal time within each sextant).

For open-field and elevated-plus maze tests, differences were analyzed by one-way ANOVA, followed by Dunnett test with Bonferroni correction.

Difference with a *p* < .05 was considered to be statistically significant.

## Results

### Study 1: Time-course of neuroinflammation evaluated by IL-1b and microglial phenotypes after TBI

#### IL-1b

**Fig 3:**
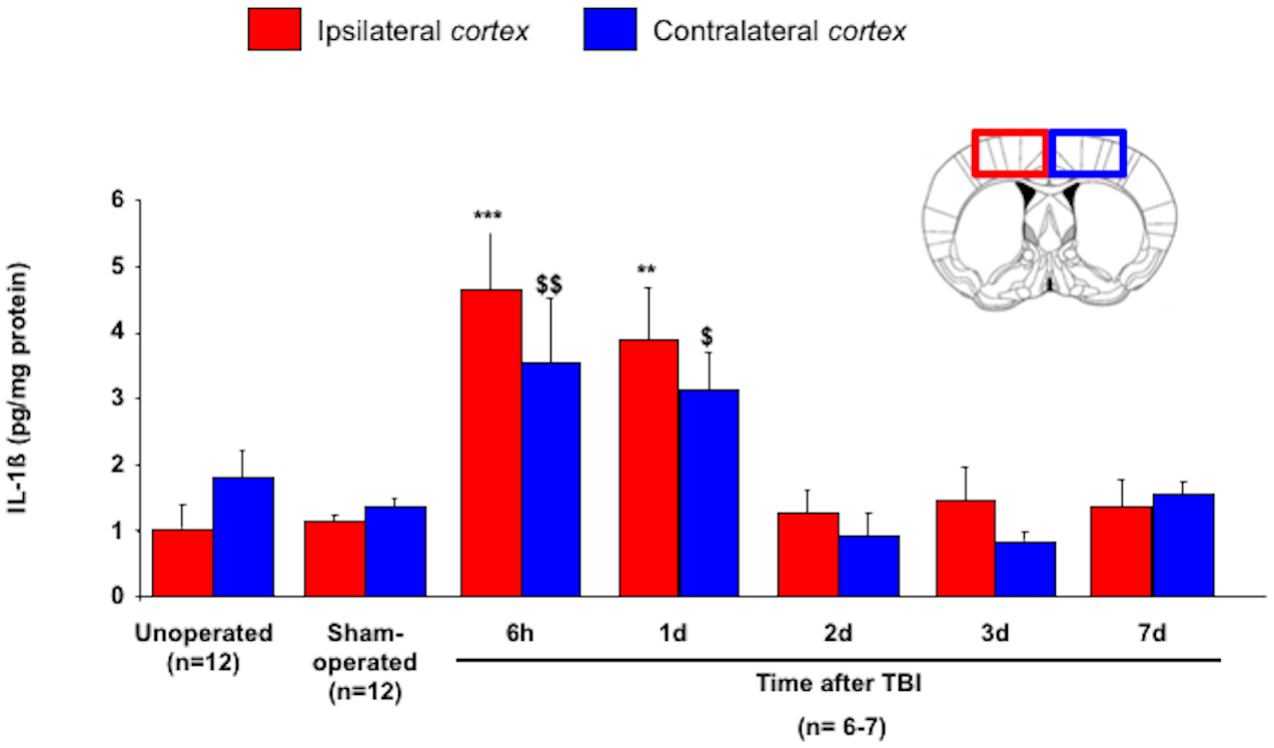
Time course of IL-1b protein in cortex after mild TBI. IL-1b protein was measured in both ipsi- (contusion) and contralateral cortex of unoperated, sham-operated and TBI mice at 6 hours, 1, 2, 3 and 7 days post-injury. Data are expressed as means ± S.E.M. Differences were analyzed using non-parametric Kruskal-Wallis test with subsequent comparison by Mann-Whitney U test. **p < 0.01 and ***p < 0.001 *vs* sham-operated ipsilateral *cortex*; $ *p* < 0.05 and $ $ *p* < 0.01 *vs* sham-operated contralateral *cortex*.

IL-1b protein levels in both ipsi- and contralateral *cortex* of unoperated (Fig 3; ipsi: 1.3±0.2pg/mg of protein; contra: 1.8±0.4pg/mg of protein) and sham-operated mice (ipsi: 1.1±0.1pg/mg of protein; contra: 1.3±0.2pg/mg of protein) were not different. TBI led to an increase in IL-1b in the ipsilateral *cortex* at 6 hours (4.6±0.9pg/mg of protein; *p*<0.001) and 1 day after TBI (3.9±0.8pg/mg of protein; *p*<0.01). Similarly, the contralateral *cortex* also revealed an increase in IL-1b at 6 hours (3.5±1.0pg/mg of protein; *p*<0.01) and 1 day after TBI (3.1±0.6pg/mg of protein; *p*<0.05). Subsequently, the level of IL-1b decreased and was not different from that of sham-operated mice from 2 to 7 days after TBI.

#### Microglial phenotypes

**Fig 4:**
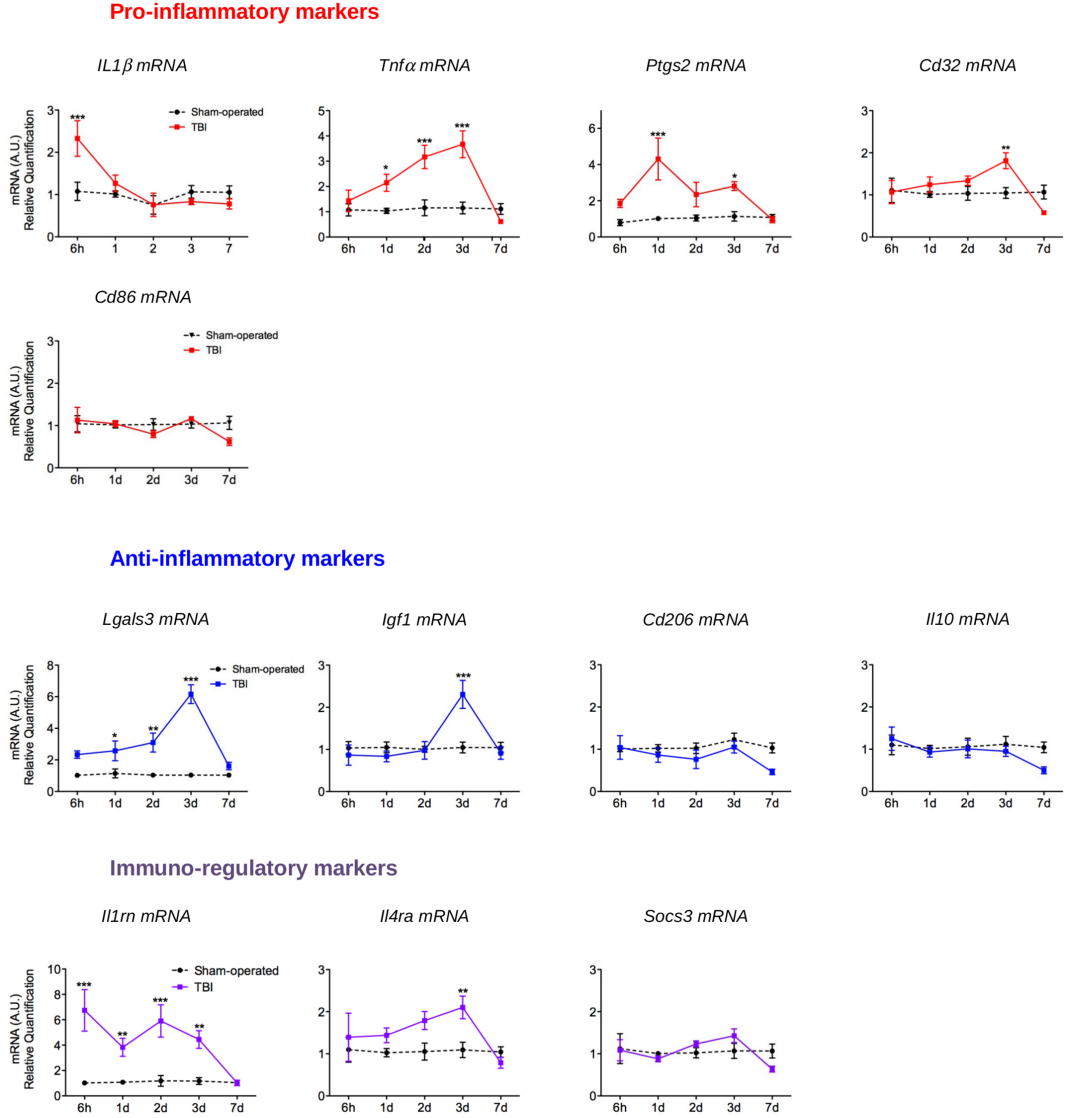
Time course of microglial phenotype after mild TBI. Quantification of pro-inflammatory (Il1b, Tnfa, Ptgs2, Cd32 and Cd86 mRNA), anti-inflammatory (Lgals3, Igf1, Cd206 and Il10 mRNA) and immunoregulatory markers (Il1rn, Il4ra and Socs3 mRNA) by RT-qPCR in CD11b^+^ cells magnetically sorted from brain of sham-operated and TBI mice at 6 hours, 1, 2, 3 and 7 days post-injury. mRNA expression was presented as a fold change relative to sham-operated mice. Data were expressed as means ± S.E.M. Differences were analyzed by 2-way ANOVA with subsequent group comparisons by Dunnett test with Bonferroni correction. * *p*<0.05, ** *p*<0.01 *** *p*<0.001 *vs* sham-operated.

Gene expression analysis for twelve validated microglial activation markers and cytokines [24] was realized to study activation of CD11b^+^ cells, from sham-operated and TBI mice, isolated by magnetic bead separation (MACS). IL1b mRNA associated with pro-inflammatory phenotype and IL1rn mRNA associated with immunoregulatory phenotype were increased at 6 hours after mild TBI (Fig 4). At 1 day, gene expression showed that TBI induced in microglia a robust pro-inflammatory phenotype characterized by a significant increase of Ptgs2 and Tnfa mRNA. At this time point, the anti-inflammatory marker Lgals 3 mRNA was also increased. Expression of markers associated with anti-inflammatory phenotype (Lgals3 and Igf1) were maximal at 3 days after injury. At this time, the overexpression of the pro-inflammatory markers Cd32 and Tnfa mRNA also reached their maximum but Ptgs2 mRNA level decreased compared to 1 day. At 7 days after injury, all markers returned to their basal levels.

### Study 2: Time course of white matter injury

#### Myelin protein expression in *corpus callosum* after TBI

**Fig 5:**
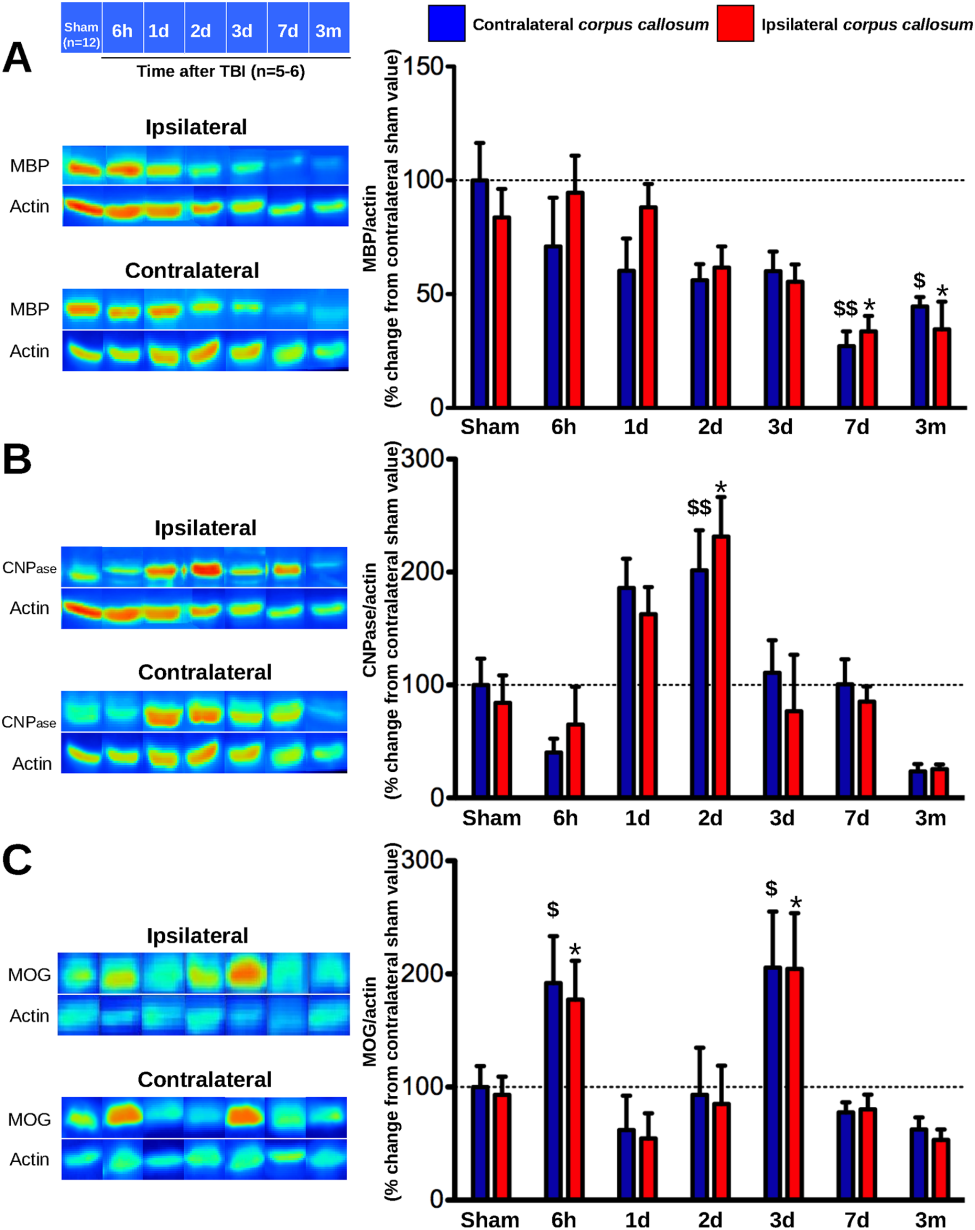
Time course of myelin proteins expression after mild TBI. (A) MBP, (B) CNPase and (C) MOG proteins expression were assessed in both ipsi- and contralateral *corpus callosum* of sham-operated and TBI mice at 6 hours, 1, 2, 3, 7 days and 3 months post-injury. Data were expressed as means ± S.E.M. Differences were analyzed using non-parametric Kruskal-Wallis test with subsequent comparison by Mann-Whitney U test. *p < 0.05 *vs* sham-operated ipsilateral *corpus callosum*; $ *p* < 0.05 and $ $ *p* < 0.01 *vs* sham-operated contralateral *corpus callosum*.

TBI decreased MBP protein expression in both ipsi- and contralateral *corpus callosum* from 7 days (Fig 5A; ipsi: *p*<0.05; contra: *p*<0.01) demonstrating a bilateral demyelination of *corpus callosum* that persisted up to 3 months post-injury (ipsi: *p*<0.05; contra: *p*<0.05).

TBI promoted a bilateral increase in CNPase protein expression in *corpus callosum* at 2 days (Fig 5B; ipsi: *p*<0.05; contra: *p*<0.01) showing an increase in immature and mature myelinating OL in both ipsi- and contralateral *corpus callosum* as an adaptative response to injury.

TBI promoted a bilateral increase in MOG in *corpus callosum* at 6 hours (Fig 5C; ipsi: *p*<0.05; contra: *p*<0.05) and at 3 days (ipsi: *p*<0.05; contra: *p*<0.05).

### Ultrastructural changes of fibers in the *corpus callosum* after TBI

**Fig 6:**
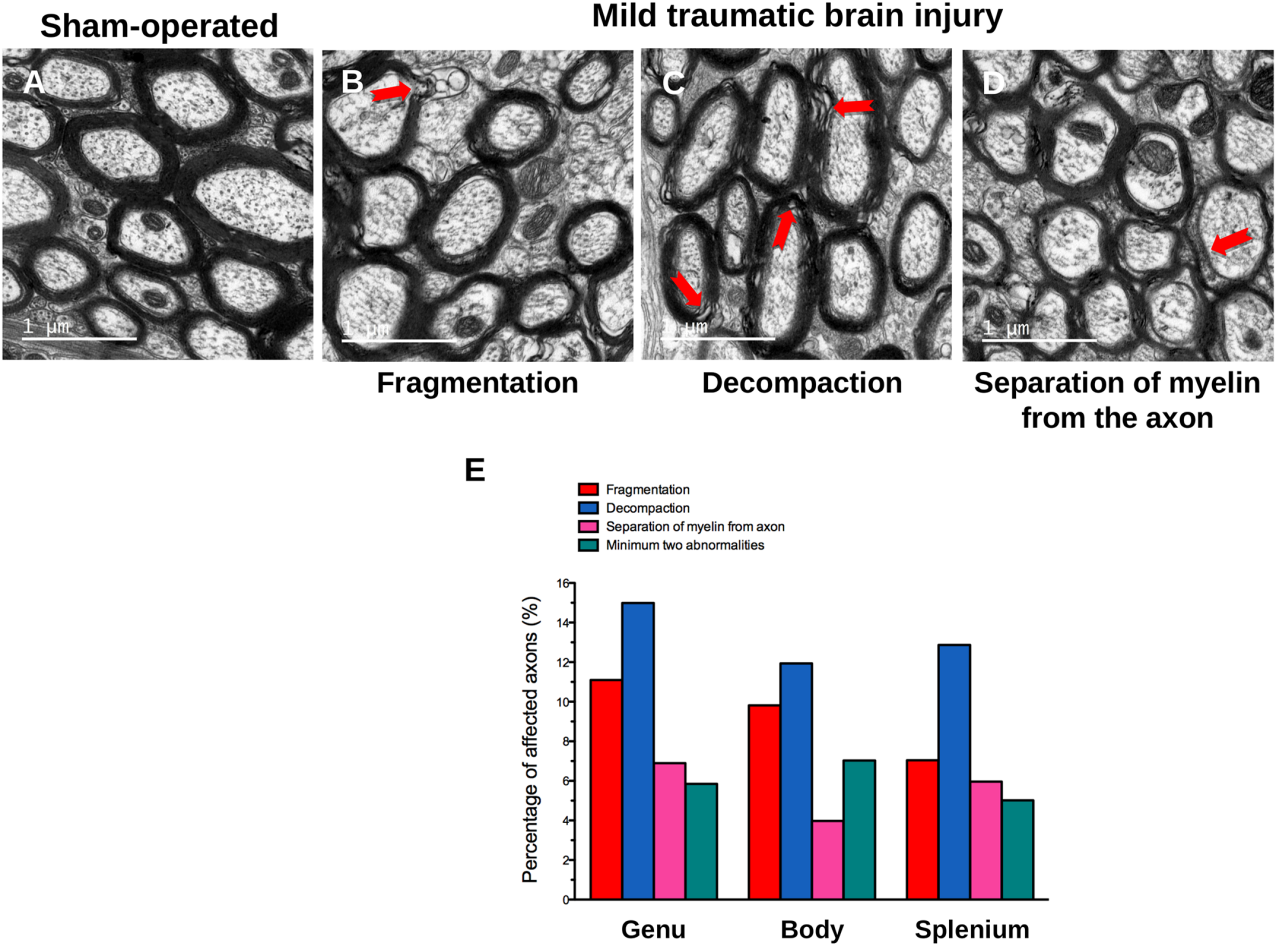
Representative electron micrographs of the myelin sheath abnormalities after mild TBI. Myelin sheath abnormalities in (A) sham-operated and (B, C, D) after mild TBI and (E) their quantification in genu, body and splenium of *corpus callosum* at 3 months after TBI. Scale bar represents 1 μm. Abnormalities were presented as percentage of affected axons after TBI.

Representative electron micrographs of the *corpus callosum* from all studied regions (genu, body and splenium) are shown in (Fig 6A–6D). Sham-operated mice showed myelinated axons with thick myelin sheath (Fig 6A). These axons were myelinated by normal intact sheath. Myelinated axons of TBI mice exhibited various abnormalities namely fragmentation (Fig 6B) or decompaction of the myelin sheath (Fig 6C), or separation of myelin from the axon (Fig 6D). Quantification of these latter showed that sham-operated presented abnormalities in axons of genu (fragmentation: 6%; decompaction: 9%; separation: 4%; minimum 2 abnormalities: 1%), body (fragmentation: 9%; decompaction: 11%; separation: 3%; minimum 2 abnormalities: 3%) and splenium (fragmentation: 8%; decompaction: 13%; separation: 4%; minimum 2 abnormalities: 3%) that may originate from surgery and the fact that myelin is prone to artifacts due to fixation and dehydration. These abnormalities were increased after mild TBI, particularly axons with at least two abnormalities in the genu (5% of affected axons *versus* 1% in sham-operated), body (7% of affected axons *versus* 3% in sham-operated) and splenium (6% of affected axons *versus* 3% in sham-operated; Fig 6E).

### Study 3: Time course of behavioral deficits after TBI

#### Sensorimotor, locomotor, exploration and defense behavior

**Fig 7:**
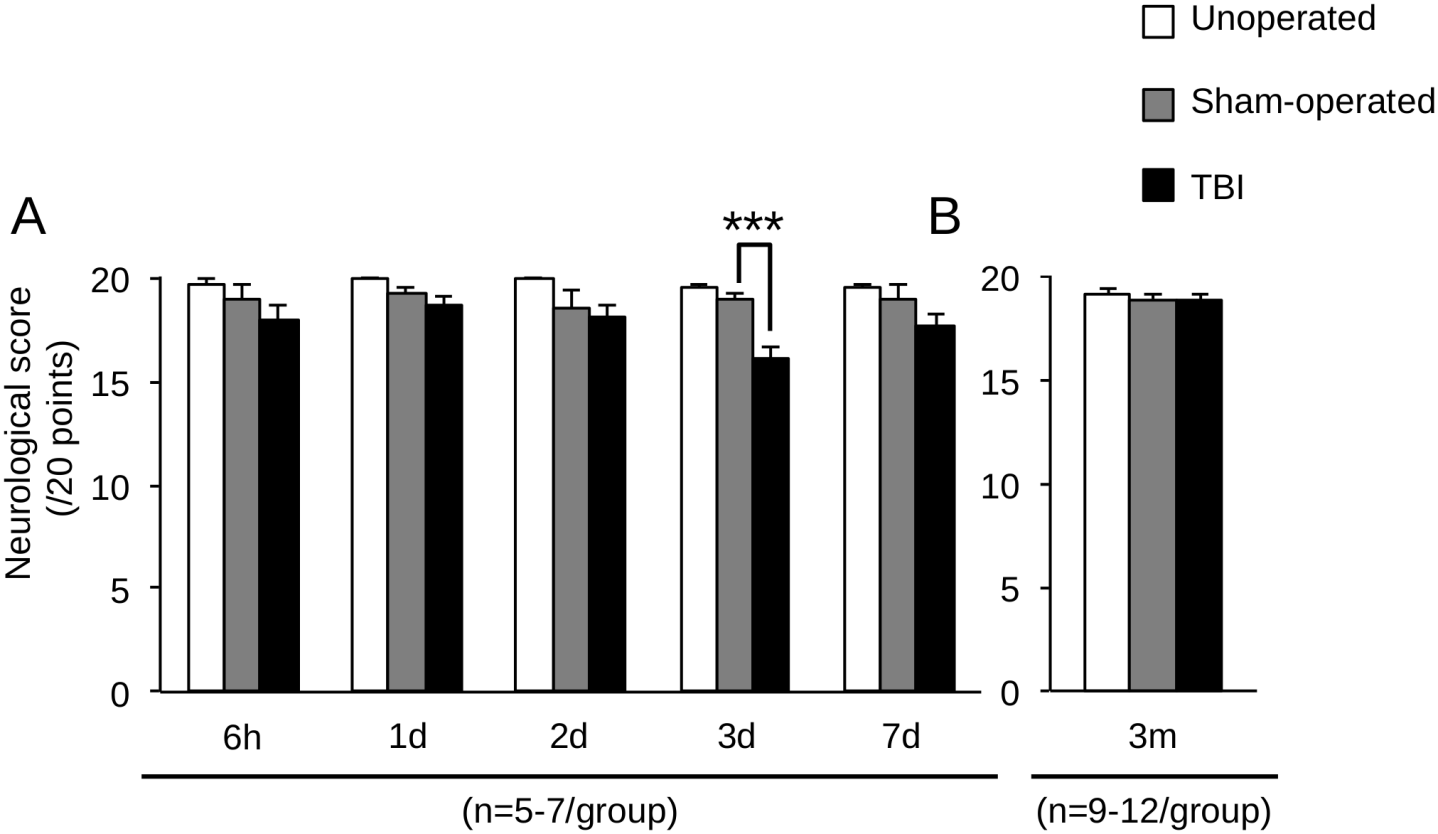
Time course of sensorimotor performances after mild TBI. Sensorimotor performances were assessed by neurological score on unoperated, sham-operated and TBI mice at (A) 6 hours, 1, 2, 3 and 7 days, and (B) 3 months. Data were expressed as means ± S.E.M. Differences were analyzed by 2-way ANOVA for repeated measures, followed by a Dunnett test with Bonferroni correction. ***p < 0.001.

Sensorimotor performances, evaluated by the neurological score, were not modified by surgery from 6 hours to 7 days (Fig 7A), and at 3 months (Fig 7B; unoperated: 19.1±0.3; sham-operated: 18.9±0.3). TBI induced a transient sensorimotor deficit at 3 days (Fig 7A; 16.2±0.5, *p*<0.001 *vs* sham-operated).

**Fig 8:**
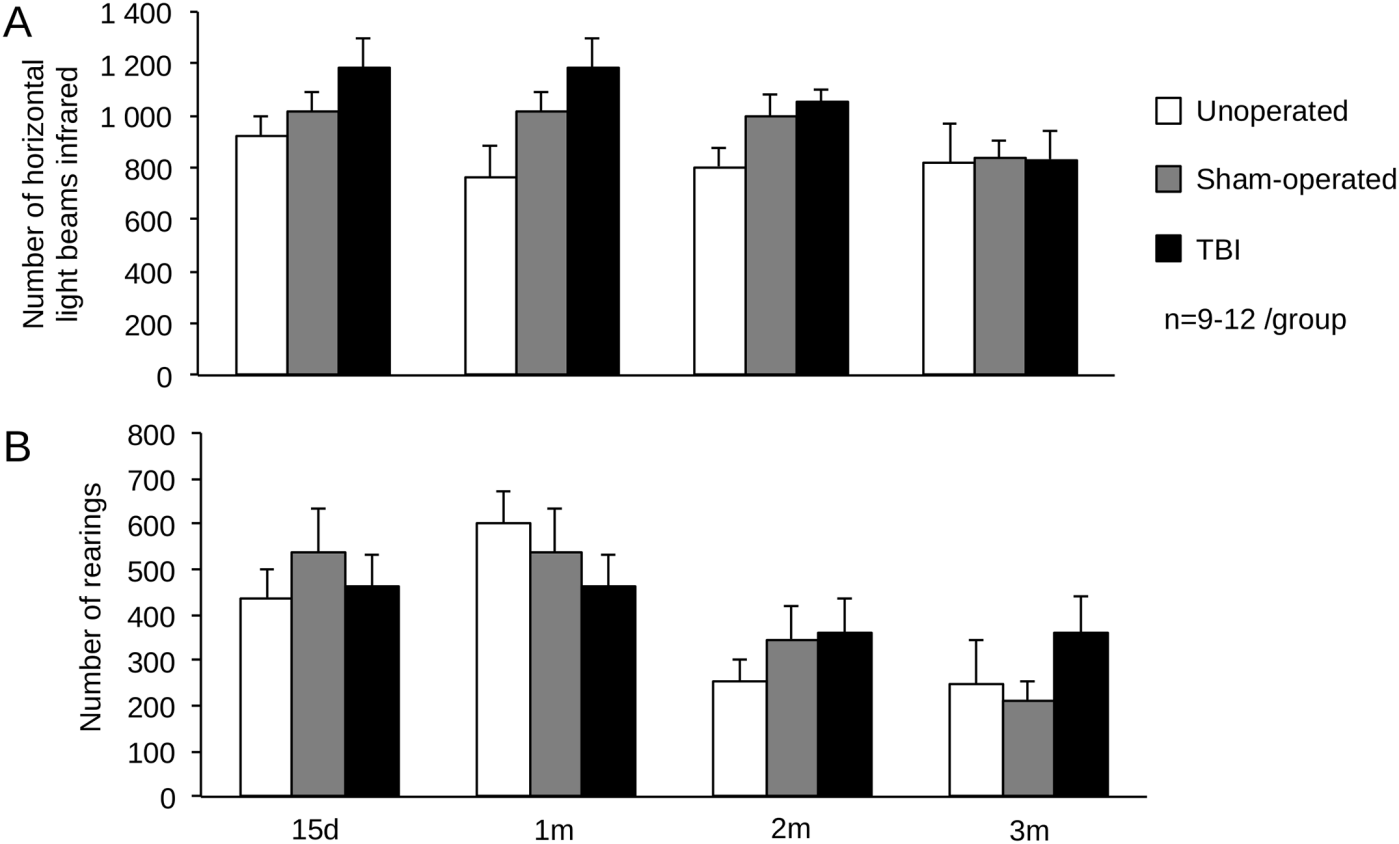
Time course of locomotor spontaneous activity after mild TBI. Locomotor spontaneous activity was assessed at 15 days, 1, 2 and 3 months on unoperated, sham-operated and TBI mice. (A) Spontaneous locomotor activity has been represented by the number of horizontal events, and (B) spontaneous exploration activity by the number of rearings. Data were expressed as means ± S.E.M. Differences were analyzed by 2-way ANOVA for repeated measures.

During actimetry test, the spontaneous locomotor activity, evaluated by the number of infrared horizontal light beams, did not differ between groups from 15 days to 3 months (Fig 8A). Moreover, the exploration behavior, evaluated by the number of rearings, did not differ between groups from 15 days to 3 months (Fig 8B).

**Fig 9:**
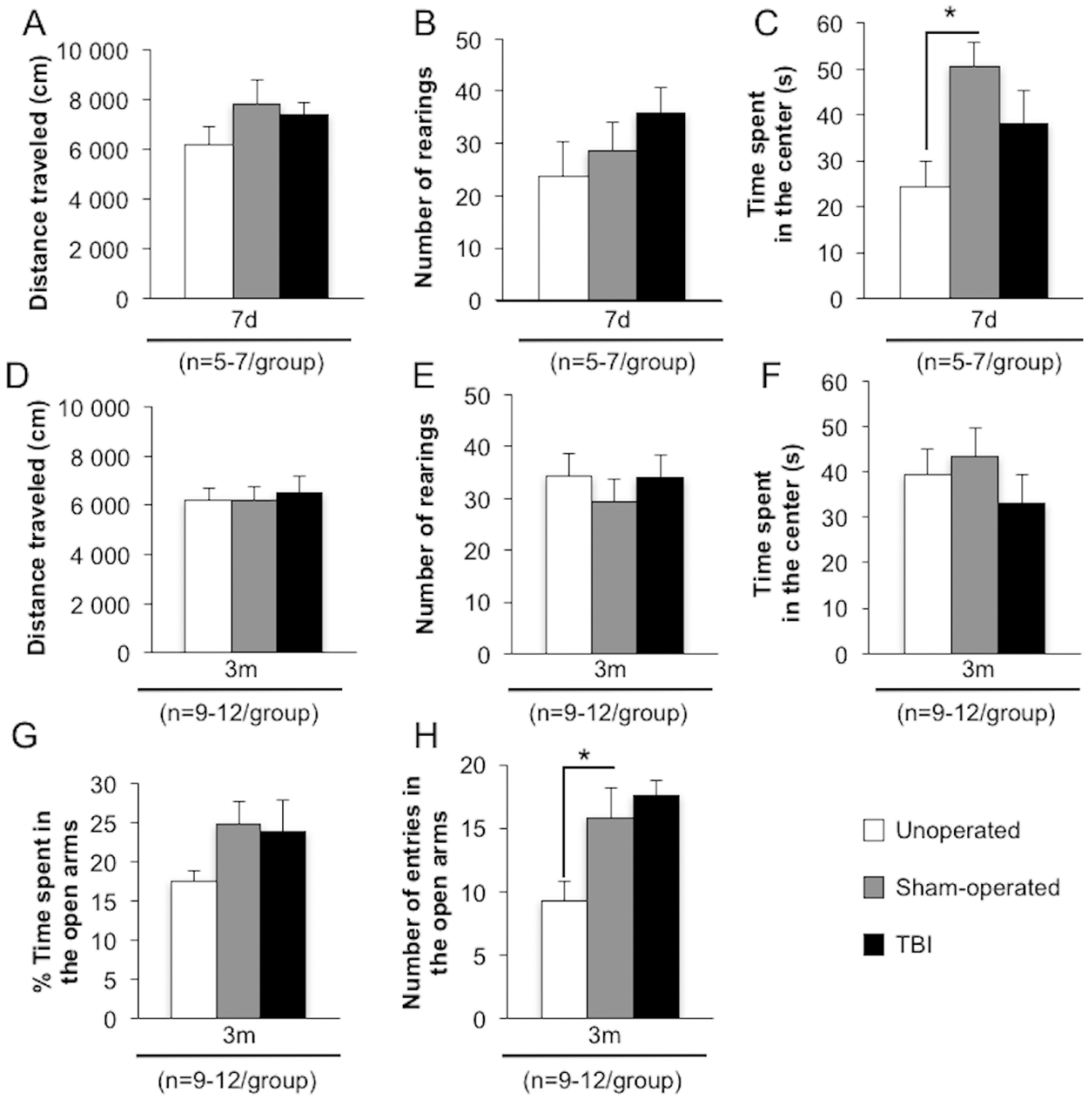
Time course of locomotor, exploration and defense behavior after mild TBI. Locomotor, exploration and defense behavior, evaluated by data collected during the open-field and the elevated-plus maze tests, were assessed on unoperated, sham-operated and TBI mice at 7 days (A, B and C) and 3 months post-injury (D, E, F, G and H). (A) represented the distance traveled (cm) at 7 days and (D) at 3 months. (B) represented the number of rearings at 7 days and (E) at 3 months. (C) represented the time spent in the center of the open-field (s) at 7 days and (F) at 3 months. (G) represented the time spent (%) in the open arms at 3 months and (H) the number of entries in the open arms of the elevated-plus maze at 3 months. Data were expressed as means ± S.E.M. Differences were analyzed by one-way ANOVA, followed by Dunnett test with Bonferroni correction. *p < 0.05 *vs* sham-operated.

Locomotor activity, evaluated by the distance traveled in the open-field, did not differ between groups at 7 days (Fig 9A) and 3 months (Fig 9D). Exploration behavior, evaluated by the number of rearings during the open-field test did not differ between groups at 7 days (Fig 9B) and 3 months (Fig 9E).

Defense behavior, evaluated by the time spent in the center of the open-field, was enhanced by surgery at 7 days (Fig 9C; unoperated: 24.4±5.3s *vs* sham-operated: 50.7±5.2s, *p*<0.05) but not by TBI (38.2±7.2s). This difference was not present at 3 months (Fig 9F; unoperated: 39.5±5.6s; sham-operated: 43.4±6.3s; TBI: 33.0±6.5s).

Furthermore, at the elevated-plus maze test, performed at 3 months, while the time spent in the open arms was not different between groups (Fig 9G), the number of entries in the open arms was increased in sham-operated mice (Fig 9H; unoperated: 9±2 *vs* sham-operated: 16±2, *p*<0.05).

#### Cognition: Spatial learning, memory and cognitive flexibility

**Fig 10:**
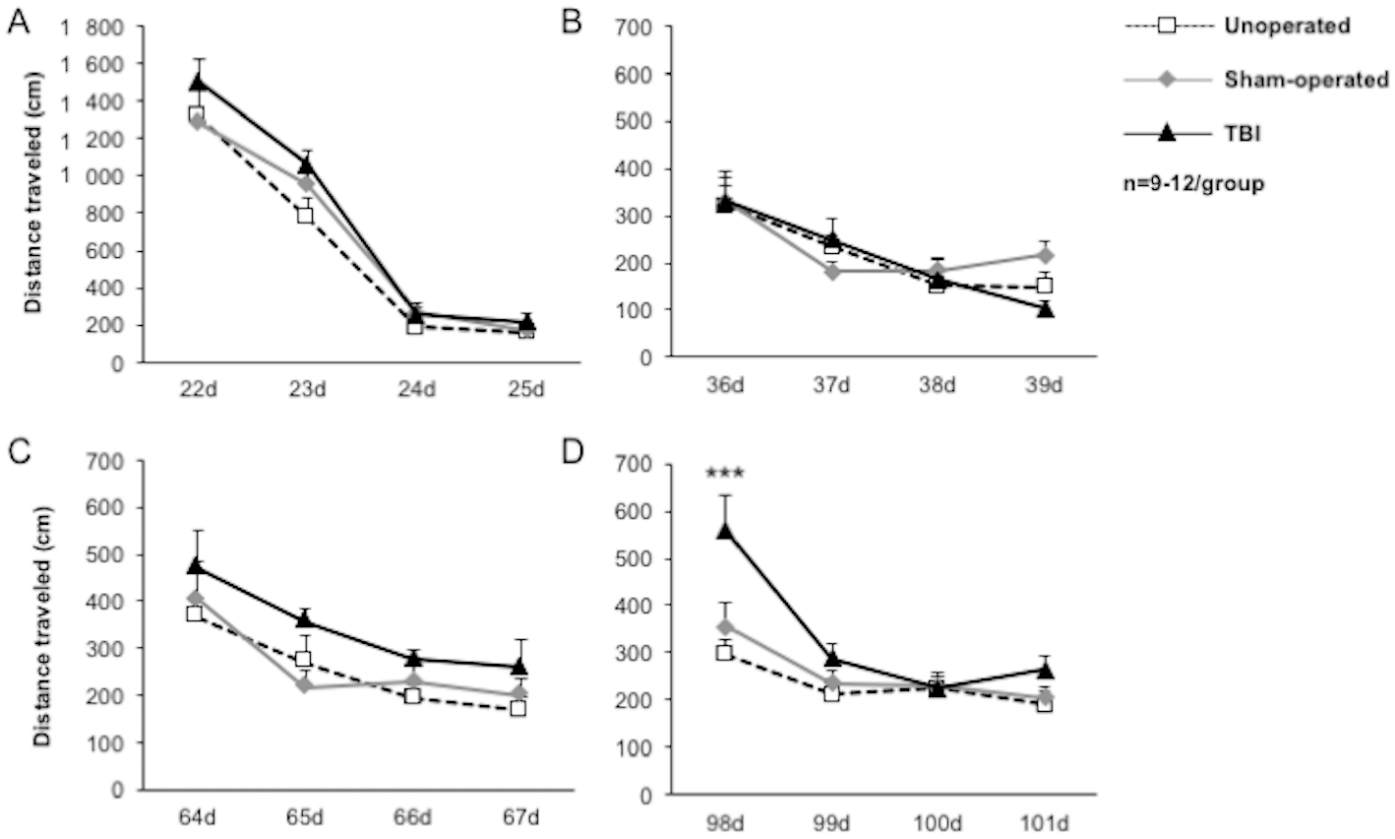
Time course of spatial learning and cognitive flexibility after mild TBI. Spatial learning and cognitive flexibility were evaluated by the distance traveled during the Barnes maze test, performed on unoperated, sham-operated and TBI mice at (A) 15 days, (B) 1 month, (C) 2 months and (D) 3 months post-injury. Data were expressed as means ± S.E.M. Differences were analyzed by 2-way ANOVA for repeated measures, followed by a Dunnett test with Bonferroni correction. ***p < 0.001 *vs* sham-operated.

At the Barnes maze test, during the learning of the first location of the escape box, the distance traveled to reach the escape box decreased from day 22 to day 25 after injury (Fig 10A; time effect *p*<0.001), with no difference between groups (group effect *p* = 0.3604, interaction *p* = 0.6287), suggesting that spatial learning was not altered by surgery or TBI. The same results were observed during the following reversal learning from day 36 to 39 (Fig 10B, time effect *p*<0.001, group effect *p* = 0.8740, interaction *p* = 0.1399) and from day 64 to 67 (Fig 10C, time effect *p*<0.001, group effect *p* = 0.2847, interaction *p* = 0.8535), demonstrating that neither surgery nor TBI induced spatial learning or cognitive flexibility deficits up to 2 months after surgery.

Interestingly, TBI induced cognitive flexibility deficit at 3 months. Indeed, mice that underwent TBI showed an increased distance traveled compared to sham-operated mice at day 98 (Fig 10D; time effect *p*<0.001, group effect *p* = 0.0047, interaction *p* = 0.0113; sham-operated: 355±50cm; TBI: 559±74cm; ***p<0.001 *vs* sham-operated). Thereafter, from day 99 to 101, learning of TBI mice was not different from sham-operated mice.

These results showed that mild TBI induced cognitive flexibility deficit at 3 months.

The number of errors (S1 Fig) and the latency to reach the escape box (S2 Fig) demonstrated the same deficit in TBI mice at 3 months.

**Fig 11:**
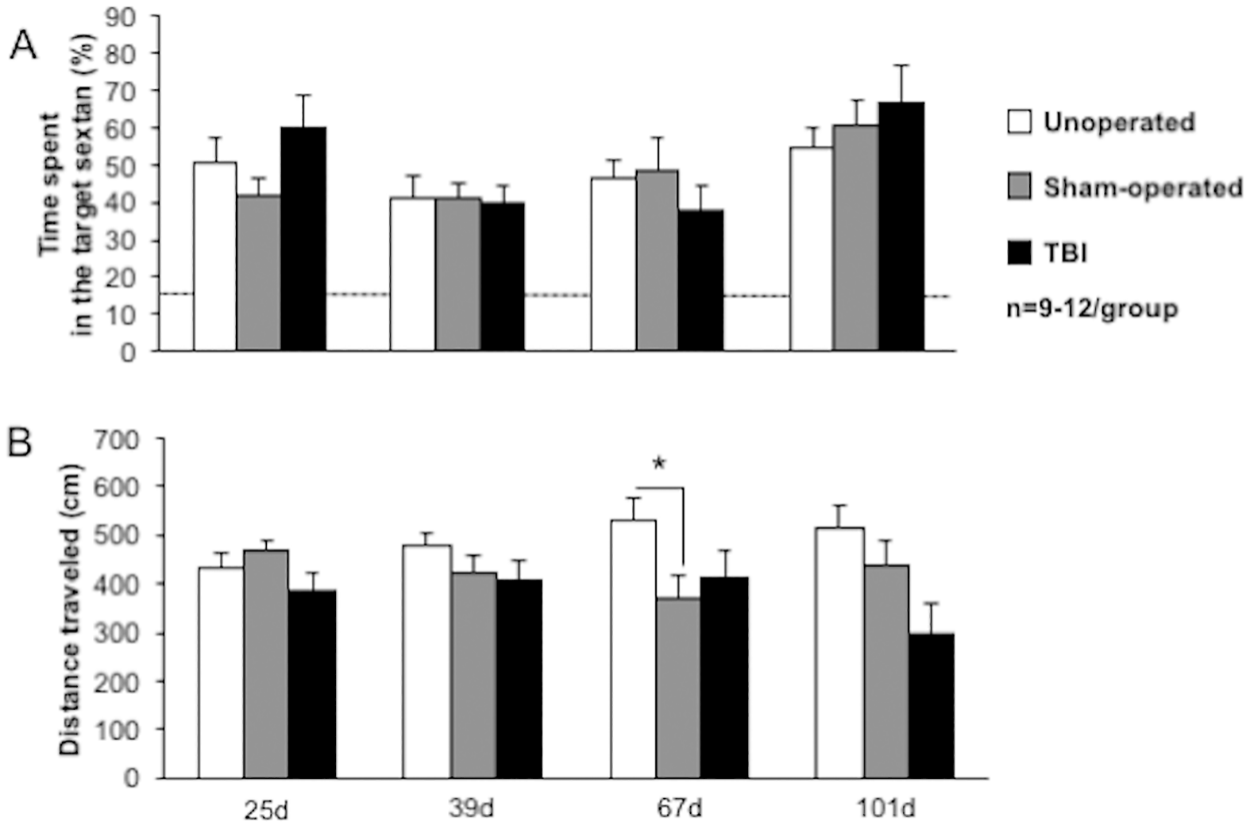
Time course of spatial retention memory performances after mild TBI. Spatial retention memory was evaluated during the probe trial in the Barnes maze, performed on unoperated, sham-operated and TBI mice with (A) the percent time spent in the target sextant and (B) the distance traveled. Data were expressed as means ± S.E.M. Differences were analyzed with univariate t test in order to compare the % time spent in the target sextant to the theoretical value 16.67% (dotted line; i.e. when the mouse spent equal time within each sextant). *p < 0.05 *vs* sham-operated.

During probe trials performed at the end of learning and reversal learnings, the percent time spent in the target sextant did not differ between groups at day 25, 39, 67 and 101 (Fig 11A; time effect *p* = 0.0027, group effect *p* = 0.5329, interaction *p* = 0.3727). Moreover, each group of mice discriminated the target sextant from the others, as the time spent in the target sextant were higher than 16.67% theoretical value, demonstrating spatial retention memory. The distance traveled during probe trials, assessing the exploration behavior during the retention task, did not differ at day 25, day 39 and day 101 (Fig 11B). Sham-operated mice showed a decreased distance traveled compared to unoperated mice (unoperated: 532±42cm; sham-operated: 369±46cm; *p<0.05).

## Discussion

Human TBI results in both neuroinflammation and WMI [9,45]. In addition, WMI contributes to cognitive and emotional disorders in traumatized patients [46]. Neuroinflammation is a major pathological process in the secondary response following injury. The inflammatory process is characterized by cellular and molecular responses respectively mediated notably by microglia activation and IL-1b secretion. Thus, neuroinflammation was evaluated by IL-1b expression and microglial activation. The latter was investigated by determining the both classical (pro-inflammatory, M1-like) and alternative activation (repair and regeneration, immunomodulatory, M2-like). IL-1b expression was increased from 6 hours to 1 day post-injury, as previously described in a severe closed skull model of TBI [36,47,48]. IL-1b is an inflammatory cytokine that participates to microglial activation, and is also produced by themselves. The time course of microglia phenotypes was determined by studying gene expression, using mRNA extracted from microglia isolated from the whole brain by the magnetic sorting technique. To date, microglial activation has been studied using markers of microglia phenotypes using FACS [32] and immunostaining [30,32]. Two studies evaluated gene expression of pro-inflammatory, anti-inflammatory and immunomodulatory markers on brain, without sorting any kind of cells, thus reflecting entire brain inflammation [32,49]. This demonstrates that these previous works did not know the cellular origin of post-traumatic inflammation. Here, we demonstrated that both pro-inflammatory and immunoregulatory phenotypes were present and last up to 3 days. The anti-inflammatory phenotype was present at 2 and 3 days. Thus, mixed microglia phenotypes were present early after injury, particularly at 3 days after mild TBI. At 7 days, there was no longer activation of microglia. Surprisingly, the *il1b* mRNA, a key pro-inflammatory mediator, was increased only at 6 hours after TBI, whereas the protein expression was also increased at 6 hours. This could be explained, by the fact that IL-1b is not directly encoded from its gene, but generated from its inactive cytoplasmic precursor (pro-IL-1b) through cleavage by caspase-1 [50]. It could be hypothesized that a mild TBI promotes a release of active IL-1b at 6 hours by the activation of caspase-1, without initiating transcription of the gene, very early after TBI. Moreover microglia could not be the source of IL-1b at this time point. The latter can originate from others cells including astrocytes, endothelial cells, neurons and oligodendrocytes [51]. Interestingly, gene expression of IL-1rn (also called IL-1ra), a competitive antagonist of IL-1r (the receptor of IL-1b), is increased from 6 hours to 3 days post-injury. The binding of IL-1rn onto IL-1r blocks all known action of IL-1b [50]. So, activated microglia, could also block IL-1b protein actions through the expression of IL-1rn. This could, somehow, explain the short-term activation of microglia after mild TBI. The present study, as well as previous reports, showed the presence of a mixed population of microglia with both pro-inflammatory, anti-inflammatory and immunoregulatory properties after TBI [29–32,49]. As expected, the temporal evolution of microglia phenotypes varies according to the severity of TBI. In a more severe TBI model of CCI, both pro- and anti-inflammatory phenotypes were expressed early after TBI, but the transient up-regulation of anti-inflammatory phenotype was replaced by pro-inflammatory and “Mtransitional” (expressing both M1-like and M2-like markers) phenotypes 7 days post-injury [32]. Others described, by immunohistochemistry, that a transient M2-like response was replaced by a chronic M1-like response in a more severe model. In addition, the M1-like response was correlated with the extent of WMI [30]. Using the same parameters of impact as those used in our study, recent data showed that the microglial response was not so clearly delineating in CCI model [49]. However, in this study, RT-qPCR analyses were performed on cortical tissue without CD11b^+^ selection demonstrating that the extracted mRNA was originated from all brain cells, without any specificity to microglia. In our study, M1-like and M2-like markers are present simultaneously from 1 to 3 days. One could hypothesize that they are present in the same CD11b^+^ cell. In the future, it will be interesting to evaluate the Mtransitional microglia phenotype after mild TBI with this model. Interestingly Wang and colleagues [30] demonstrated that conditioned medium from M1-like microglia exacerbated oligodendrocyte death induced by oxygen glucose deprivation, while cell death was alleviated by conditioned medium from M2-like microglia. This suggests that maintaining the anti-inflammatory microglia phenotype could therefore benefit the traumatized brain, particularly through protective effect on WMI.

WMI can be evidenced by demyelination, through the level of myelin proteins promoting the structure of the myelin sheath. To study these alterations three different markers, MBP, CNPase and MOG protein expression were evaluated in *corpus callosum*. This structure was chosen as it is the largest white matter tract in the brain and clinical data emphasized its vulnerability following TBI [52]. MBP is a protein involved in the myelin sheath formation that has been also used as a biomarker of injury severity in both pediatric and adult TBI [53,54]. The 21.5 kDa MBP isoform was evaluated as previous studies demonstrated the vulnerability of this isoform following WMI [37]. Our results demonstrated a TBI-induced decrease in MBP expression in both ipsi- and contralateral *corpus callosum* starting at 7 days up to 3 months post-injury, demonstrating that TBI led to a bilateral demyelination that takes place away from the site of impact. Consistent with our results, others have showed a decrease of MBP immunostaining and an abundant SMI32 staining, a known marker of axonal damage, in the *corpus callosum* at 3 and 7 days post-TBI [30] and 12 months after single mild [17] or moderate TBI [23]. As TBI leads to calpain and matrix metalloproteinase 9 (MMP9) activation [4], decrease in MBP expression may originate from its proteolysis by calpain [55] or MMP9 activation [37].

A second myelin marker, CNPase, expressed in both immature and mature OL, has been chosen as this protein is generally considered a marker of myelin-producing cell rather than a marker of myelination [56]. Samples used for MBP protein expression study were then assessed for CNPase protein expression. TBI promoted an increase in CNPase expression in both ipsi- and contralateral *corpus callosum* at 2 days post-injury, suggesting an increase in myelin-producing cells in response to TBI. An increase in Olig2, a marker of both immature and mature OL, was described at 2 and 7 days after TBI in adult mice [14,15], while others did not reveal any CNPase modification following CCI in juvenile mice [57]. Moreover, proliferative Olig2^+^ cells were also reported in *corpus callosum* within 2 days after injury, further increased at 1 and 2 weeks, with lower cell numbers at 3 months post-TBI. In severe focal human TBI, OL death and increases in OPC post-injury were described [16]. It could be hypothesized that MBP alteration initiating from 1–2 days, although not statistically reported, might promote an attempt by the brain to compensate for the beginning demyelination, as it has been observed in brain of vascular dementia patients [58] and during peak of multiple sclerosis [59].

Unlike MBP and CNPase, MOG is quantitatively a minor myelin protein present in the later stages of myelination [39,60]. MOG is not present in peripheral nervous system that also contains myelin, suggesting that MOG has other possible functions than just participation in myelin formation. Indeed, although the exact role of MOG is unclear, three possible functions of MOG are proposed: a myelin sheath adhesion molecule [39], a regulator of oligodendrocyte microtubule stability [59], and a mediator of interaction between myelin and the immune system through the direct activation of the classical pathway of the complement, in particular the C1q component. The latter role is supported by its position on the extracellular surface of non-compact myelin [39]. Injection of a specific fragment of MOG is used to promote experimental autoimmune encephalomyelitis in mice in order to reproduce white matter lesions described in multiple sclerosis, suggesting that MOG can play a deleterious role in demyelinating diseases [61]. As there was no data about MOG protein expression after TBI, we studied its time course following injury. We showed a bilateral increase in MOG at 6 hours and 3 days, suggesting that the classical pathway of the complement, in which MOG participates, may be activated in order to protect myelin from TBI. Thus, the increase in MOG protein expression may trigger anti-MOG antibody production, and the subsequent formation of the MOG/anti-MOG complex, which in turn may lead to MBP degradation. In order to test whether TBI led to an increased MOG protein expression that could further contribute to anti-MOG autoantibody production, the latter was measured in CSF and serum of mice 2 months after TBI, a time-point that could be sufficient to the immune machinery to produce antibodies. However, we did not detect anti-MOG autoantibody after TBI, demonstrating that TBI did not lead to an autoimmune-mediated demyelination. However more precise examination of the MOG expression and autoimmunity mechanism need to be performed.

All these data suggested that there are two phases after TBI induced by CCI. The first phase, starting from 6 hours up to 3 days post-injury, is characterized by an increase in CNPase and MOG protein expression, reflecting respectively an increase in myelin-producing cells, and a possible activation of immune system, which may be induced in response to brain injury in order to protect from myelin loss. The second phase, from 7 days up to, at least, 3 months post-injury, is characterized by a decrease in MBP protein expression probably due to i) its degradation by calpains and MMP9 and ii) the failure of events in the first phase.

In order to evaluate the impact of myelin protein loss on the myelin sheath morphology in the *corpus callosum*, we conducted a TEM study at 3 months, since MBP loss was still present at 3 months after TBI. Three regions of the *corpus callosum*, genu, body, and splenium, had been studied. Within each callosal region, we have showed that mild TBI results in various myelin alterations including fragmentation and decompaction of the myelin sheath as well as separation of myelin from axon, suggesting a decrease in WM integrity. These abnormalities were observed separately in different myelinated axons, but were simultaneously present within the same myelinated axons 3 months after TBI. Similar ultrastructural alterations had been previously described 2 months after TBI induced by CCI in rat [62]. Abnormal myelin sheath gaps might result in slowing of the information-processing speed, which is seen in TBI patients [63,64]. Our results reflect morphological alterations in all callosal regions although some parts of *corpus callosum* were not underneath the impact site. This confirms that TBI, albeit mild, might lead to demyelination that can take place away from the site of impact.

Demyelination could originate from OL cell death as demonstrated by Dent and colleagues [15]. In the latter study, TBI, also induced by CCI, promoted mature OL apoptosis in *corpus callosum*. In addition, newborn OL that differentiate into mature OL were detected and remained present at least 3 months post-injury. It is therefore hypothesized that these newly formed OL may be incapable of myelination, as the three myelin proteins expression we studied were lower at 3 months post-injury. All these data suggest that oligodendrogenesis induced by TBI does not permit remyelination.

TBI-induced demyelination, here evidenced by MBP decrease and ultrastructural abnormalities of myelin sheath, could be 1) primary, which means demyelination of intact axons, or 2) subsequent to the focal axons loss caused by haemorrhage or neuron cell death along with axon degeneration or 3) both at the same time. Moreover, as myelin contributes to the regulation of the axonal cytoskeleton and caliber, myelin disturbances might cause axonal disturbances due the alteration of the axon-myelin relationship [62]. Our results showed myelin abnormalities within myelin sheath surrounding axons. But, we have no data available to exclude/argue a loss of myelin due to axonal loss. However, previous study [11] using the same CCI parameters as we used, observed axonal injury and neurodegeneration, suggesting that axonal injury could also explain, the demyelination showed in our study. Last, but not least, myelin (electron microscopy) and axonal degeneration (b-APP staining) were recently described in *corpus callosum* in a model of closed-skull impact on adult mice [18]. This study demonstrated the presence of degenerating axons among intact axons, and myelin pathologic features.

WMI is known to be responsible for neurobehavioral disorders that can develop slowly and persist for years after TBI [46,65]. Therefore, we evaluated time course of behavioral performances from 6 hours until 3 months after injury. While the actimetry and the open-field test failed to evidence spontaneous exploration behavior modifications, our data showed that TBI induced sensorimotor deficits, revealed by the neurological score, at 3 days. The neurological score used in our study, evaluating reflex and postural behavior only, has already been shown to reveal sensorimotor deficits until 1 month after a cerebral focal ischemia [40]. Using a slightly more severe TBI, however called mild by the authors, authors did not show gross locomotor function in the open-field test at 3 weeks post-injury [66]. In a more severe model of CCI, sensorimotor deficit was present for 1 month [67]. Long term sensorimotor deficit is also present in a semi-circular model of CCI, but not with a classical circular model of CCI [68], suggesting that only severe model of TBI could induce long term sensorimotor deficits.

Another finding of our study is the modification of defense behaviors in the open-field and the elevated-plus maze test induced by the surgical procedure. It is now suggested in the literature that craniotomy itself could induce inflammation and edema [69]. Last but not the least, our unconditioned behavioral tests (neurological score, actimetry, open-field and elevated-plus maze) are able to reveal severe deficits of spontaneous behaviors, but could be insufficient to reveal complex behavioral disorders, involving integrative processes dependent of WMI, in this mild model of TBI.

Originally used in rat models of TBI with training before injury [70] or after injury [71], the Barnes maze has shown its ability to reveal spatial learning and memory deficits, and modification of research strategy [72] in models of moderate to severe TBI. The Barnes maze revealed spatial learning deficits 1 month after moderate to severe CCI [73]. In our study, although it failed to evidence any spatial learning and memory deficit, a late cognitive flexibility disorder, using reversal learning, was evidenced at 3 months after TBI. To our knowledge, no study has used the reversal learning in a spatial task after TBI in rodents to reveal any cognitive flexibility disorders. All other studies were performed earlier than ours. In a similar task using the Morris water maze, it has been shown that moderate or severe, but not mild, CCI induced spatial learning deficits in classical and reversal learning 2 weeks after the insult, without any cognitive flexibility disorder [74]. The latter has been shown, using the rule shift assay, 40 days after a specific prefrontal cortex insult by CCI [75].

Some attentional deficits have been observed at 3 weeks after TBI [76]. Attention and flexible cognitive control has been described to be dependent on interactions within the fronto-striato-thalamic circuit in humans. The deficit of flexibility observed in TBI patients is closely related to subcortical atrophy of this circuit which is closely related to white matter microstructural modifications [77]. Thus, our study highlights that cognitive flexibility disorder induced by mild TBI, without any learning and memory deficits, appears at long term after injury and could result from WMI observed in the acute phase after mild TBI.

In conclusion, using this mouse model of mild TBI, our study affords a significant comprehensive overview of neuroinflammation, demyelination and cognitive flexibility disorders during the acute and chronic phase traumatic-induced mild brain injury. This model might serve for exploring molecular and cellular target to further propose pharmacological strategies aiming at reducing mild TBI consequences.

## Supporting information

S1 Fig Time course of spatial learning and cognitive flexibility, evaluated by the number of errors, after mild TBI. The number of errors was obtained during the Barnes maze test, performed on unoperated, sham-operated and TBI mice at (A) 15 days, (B) 1 month, (C) 2 months and (D) 3 months post-injury. Data were expressed as means ± S.E.M. Differences were analyzed by 2-way ANOVA for repeated measures, followed by a Dunnett test with Bonferroni correction. *p < 0.05 *vs* sham-operated.
(TIF)

S2 Fig Time course of spatial learning and cognitive flexibility, evaluated by the escape latency, after mild TBI. The escape latency was obtained during the Barnes maze test, performed on unoperated, sham-operated and TBI mice at (A) 15 days, (B) 1 month, (C) 2 months and (D) 3 months post-injury. Data were expressed as means ± S.E.M. Differences were analyzed by 2-way ANOVA for repeated measures, followed by a Dunnett test with Bonferroni correction. *p < 0.05 *vs* sham-operated.
(TIF)

## References

[1] Maas AIR, Stocchetti N, Bullock R. Moderate and severe traumatic brain injury in adults. Lancet Neurol. 2008; 7(8):728–41. doi: 10.1016/S1474-4422(08)70164-9 18635021 10.1016/S1474-4422(08)70164-9

[2] Levin HS, Robertson CS. Mild traumatic brain injury in translation. J Neurotrauma. 2013; 30(8):610–7. doi: 10.1089/neu.2012.2394 23046349 10.1089/neu.2012.2394 PMC3638686

[3] Kou Z, VandeVord PJ. Traumatic white matter injury and glial activation: from basic science to clinics. Glia. 2014; 62(11):1831–55. doi: 10.1002/glia.22690 24807544 10.1002/glia.22690

[4] Büki A, Povlishock JT. All roads lead to disconnection?—Traumatic axonal injury revisited. Acta Neurochir (Wien). 2006;148(2):181–193; discussion 193–194. 16362181 10.1007/s00701-005-0674-4

[5] Scheid R, Walther K, Guthke T, Preul C, von Cramon DY. Cognitive sequelae of diffuse axonal injury. Arch Neurol. 2006; 63(3):418–24. doi: 10.1001/archneur.63.3.418 16533969 10.1001/archneur.63.3.418

[6] Arfanakis K, Haughton VM, Carew JD, Rogers BP, Dempsey RJ, Meyerand ME. Diffusion tensor MR imaging in diffuse axonal injury. Am J Neuroradiol. 2002; 23(5):794–802. 12006280 PMC7974716

[7] Inglese M, Makani S, Johnson G, Cohen BA, Silver JA, Gonen O, et al Diffuse axonal injury in mild traumatic brain injury: a diffusion tensor imaging study. J Neurosurg. 2005; 103(2):298–303. doi: 10.3171/jns.2005.103.2.0298 16175860 10.3171/jns.2005.103.2.0298

[8] Wang JY, Bakhadirov K, Abdi H, Devous MD, Marquez de la Plata CD, Moore C, et al Longitudinal changes of structural connectivity in traumatic axonal injury. Neurology. 2011;77(9):818–26. doi: 10.1212/WNL.0b013e31822c61d7 21813787 10.1212/WNL.0b013e31822c61d7 PMC3162636

[9] Johnson VE, Stewart JE, Begbie FD, Trojanowski JQ, Smith DH, Stewart W. Inflammation and white matter degeneration persist for years after a single traumatic brain injury. Brain J Neurol. 2013; 136(Pt 1):28–42. 10.1093/brain/aws322 PMC3562078 23365092

[10] Hall ED, Sullivan PG, Gibson TR, Pavel KM, Thompson BM, Scheff SW. Spatial and temporal characteristics of neurodegeneration after controlled cortical impact in mice: more than a focal brain injury. J Neurotrauma. 2005; 22(2):252–65. doi: 10.1089/neu.2005.22.252 15716631 10.1089/neu.2005.22.252

[11] Hall ED, Bryant YD, Cho W, Sullivan PG. Evolution of post-traumatic neurodegeneration after controlled cortical impact traumatic brain injury in mice and rats as assessed by the de Olmos silver and fluorojade staining methods. J Neurotrauma. 2008; 25(3):235–47. doi: 10.1089/neu.2007.0383 18352837 10.1089/neu.2007.0383

[12] Armstrong RC, Mierzwa AJ, Marion CM, Sullivan GM. White matter involvement after TBI: Clues to axon and myelin repair capacity. Exp Neurol. 2016; 275 Pt 3:328–33. 25697845 10.1016/j.expneurol.2015.02.011

[13] Lotocki G, de Rivero Vaccari JP, Alonso O, Molano JS, Nixon R, Safavi P, et al Oligodendrocyte vulnerability following traumatic brain injury in rats. Neurosci Lett. 2011; 499(3):143–8. doi: 10.1016/j.neulet.2011.05.056 21669255 10.1016/j.neulet.2011.05.056 PMC3523350

[14] Flygt J, Djupsjö A, Lenne F, Marklund N. Myelin loss and oligodendrocyte pathology in white matter tracts following traumatic brain injury in the rat. Eur J Neurosci. 2013; 38(1):2153–65. doi: 10.1111/ejn.12179 23458840 10.1111/ejn.12179

[15] Dent KA, Christie KJ, Bye N, Basrai HS, Turbic A, Habgood M, et al Oligodendrocyte Birth and Death following Traumatic Brain Injury in Adult Mice. PloS One. 2015;10(3):e0121541 doi: 10.1371/journal.pone.0121541 25798924 10.1371/journal.pone.0121541 PMC4370677

[16] Flygt J, Gumucio A, Ingelsson M, Skoglund K, Holm J, Alafuzoff I, et al Human Traumatic Brain Injury Results in Oligodendrocyte Death and Increases the Number of Oligodendrocyte Progenitor Cells. J Neuropathol Exp Neurol. 2016; 75(6):503–15. doi: 10.1093/jnen/nlw025 27105664 10.1093/jnen/nlw025

[17] Mouzon BC, Bachmeier C, Ferro A, Ojo J-O, Crynen G, Acker CM, et al Chronic neuropathological and neurobehavioral changes in a repetitive mild traumatic brain injury model. Ann Neurol. 2014; 75(2):241–54. doi: 10.1002/ana.24064 24243523 10.1002/ana.24064

[18] Mierzwa AJ, Marion CM, Sullivan GM, McDaniel DP, Armstrong RC. Components of myelin damage and repair in the progression of white matter pathology after mild traumatic brain injury. J Neuropathol Exp Neurol. 2015; 74(3):218–32. doi: 10.1097/NEN.0000000000000165 25668562 10.1097/NEN.0000000000000165 PMC4327393

[19] Finnie JW. Neuroinflammation: beneficial and detrimental effects after traumatic brain injury. Inflammopharmacology. 2013; 21(4):309–20. doi: 10.1007/s10787-012-0164-2 23296919 10.1007/s10787-012-0164-2

[20] Lozano D, Gonzales-Portillo GS, Acosta S, de la Pena I, Tajiri N, Kaneko Y, et al Neuroinflammatory responses to traumatic brain injury: etiology, clinical consequences, and therapeutic opportunities. Neuropsychiatr Dis Treat. 2015; 11:97–106. doi: 10.2147/NDT.S65815 25657582 10.2147/NDT.S65815 PMC4295534

[21] Chiu C-C, Liao Y-E, Yang L-Y, Wang J-Y, Tweedie D, Karnati HK, et al Neuroinflammation in animal models of traumatic brain injury. J Neurosci Methods. 2016; 272:38–49. doi: 10.1016/j.jneumeth.2016.06.018 27382003 10.1016/j.jneumeth.2016.06.018 PMC5201203

[22] Glushakova OY, Johnson D, Hayes RL. Delayed increases in microvascular pathology after experimental traumatic brain injury are associated with prolonged inflammation, blood-brain barrier disruption, and progressive white matter damage. J Neurotrauma. 2014; 31(13):1180–93. doi: 10.1089/neu.2013.3080 24564198 10.1089/neu.2013.3080

[23] Loane DJ, Kumar A, Stoica BA, Cabatbat R, Faden AI. Progressive neurodegeneration after experimental brain trauma: association with chronic microglial activation. J Neuropathol Exp Neurol. 2014; 73(1):14–29. doi: 10.1097/NEN.0000000000000021 24335533 10.1097/NEN.0000000000000021 PMC4267248

[24] Chhor V, Le Charpentier T, Lebon S, Oré M-V, Celador IL, Josserand J, et al Characterization of phenotype markers and neuronotoxic potential of polarised primary microglia in vitro. Brain Behav Immun. 2013; 32:70–85. doi: 10.1016/j.bbi.2013.02.005 23454862 10.1016/j.bbi.2013.02.005 PMC3694309

[25] Hernandez-Ontiveros DG, Tajiri N, Acosta S, Giunta B, Tan J, Borlongan CV. Microglia activation as a biomarker for traumatic brain injury. Front Neurol; 4:30 doi: 10.3389/fneur.2013.00030 23531681 10.3389/fneur.2013.00030 PMC3607801

[26] Bergold PJ. Treatment of traumatic brain injury with anti-inflammatory drugs. Exp Neurol. 2016; 275 Pt 3:367–80. 26112314 10.1016/j.expneurol.2015.05.024 PMC6007860

[27] Karve IP, Taylor JM, Crack PJ. The contribution of astrocytes and microglia to traumatic brain injury. Br J Pharmacol. 2016;173(4):692–702. doi: 10.1111/bph.13125 25752446 10.1111/bph.13125 PMC4742296

[28] Loane DJ, Kumar A. Microglia in the TBI brain: The good, the bad, and the dysregulated. Exp Neurol. 2016; 275 Pt 3:316–27. 26342753 10.1016/j.expneurol.2015.08.018 PMC4689601

[29] Jin X, Ishii H, Bai Z, Itokazu T, Yamashita T. Temporal Changes in Cell Marker Expression and Cellular Infiltration in a Controlled Cortical Impact Model in Adult Male C57BL/6 Mice. PLoS ONE. 2012;7(7). 10.1371/journal.pone.0041892 PMC3404031 22911864

[30] Wang G, Zhang J, Hu X, Zhang L, Mao L, Jiang X, et al Microglia/macrophage polarization dynamics in white matter after traumatic brain injury. J Cereb Blood Flow Metab Off J Int Soc Cereb Blood Flow Metab. 2013; 33(12):1864–74. 10.1038/jcbfm.2013.146 PMC3851898 23942366

[31] Turtzo LC, Lescher J, Janes L, Dean DD, Budde MD, Frank JA. Macrophagic and microglial responses after focal traumatic brain injury in the female rat. J Neuroinflammation. 2014; 11:82 doi: 10.1186/1742-2094-11-82 24761998 10.1186/1742-2094-11-82 PMC4022366

[32] Kumar A, Alvarez-Croda D-M, Stoica BA, Faden AI, Loane DJ. Microglial/Macrophage Polarization Dynamics following Traumatic Brain Injury. J Neurotrauma. 2016; 33(19):1732–50. doi: 10.1089/neu.2015.4268 26486881 10.1089/neu.2015.4268 PMC5065034

[33] Osier ND & Dixon CE. The Controlled Cortical Impact Model: Applications, Considerations for Researchers, and Future Directions. Front Neurol. 2016; 7:134 doi: 10.3389/fneur.2016.00134 27582726 10.3389/fneur.2016.00134 PMC4987613

[34] Smith DH, Soares HD, Pierce JS, Perlman KG, Saatman KE, Meaney DF, et al A Model of Parasagittal Controlled Cortical Impact in the Mouse: Cognitive and Histopathologic Effects. J Neurotrauma. 1995; 12(2):169–78. doi: 10.1089/neu.1995.12.169 7629863 10.1089/neu.1995.12.169

[35] Siopi E, Cho AH, Homsi S, Croci N, Plotkine M, Marchand-Leroux C, et al Minocycline Restores sAPPα Levels and Reduces the Late Histopathological Consequences of Traumatic Brain Injury in Mice. J Neurotrauma. 2011; 28(10):2135–43. doi: 10.1089/neu.2010.1738 21770756 10.1089/neu.2010.1738

[36] Siopi E, Llufriu-Dabén G, Cho AH, Vidal-Lletjós S, Plotkine M, Marchand-Leroux C, et al Etazolate, an α-secretase activator, reduces neuroinflammation and offers persistent neuroprotection following traumatic brain injury in mice. Neuropharmacology. 2013; 67:183–92. doi: 10.1016/j.neuropharm.2012.11.009 23178198 10.1016/j.neuropharm.2012.11.009

[37] Asahi M, Wang X, Mori T, Sumii T, Jung JC, Moskowitz MA, et al Effects of matrix metalloproteinase-9 gene knock-out on the proteolysis of blood-brain barrier and white matter components after cerebral ischemia. J Neurosci. 2001; 21(19):7724–32. 11567062 10.1523/JNEUROSCI.21-19-07724.2001 PMC6762894

[38] Radtke C, Sasaki M, Lankford KL, Gallo V, Kocsis JD, Radtke C, et al CNPase Expression in Olfactory Ensheathing Cells, CNPase Expression in Olfactory Ensheathing Cells. BioMed Res Int. 2011; e608496. 10.1155/2011/608496 PMC3228405 22174557

[39] Johns TG, Bernard CC. The structure and function of myelin oligodendrocyte glycoprotein. J Neurochem. 1999; 72(1):1–9. 9886048 10.1046/j.1471-4159.1999.0720001.x

[40] Leconte C, Tixier E, Freret T, Toutain J, Saulnier R, Boulouard M, et al Delayed hypoxic postconditioning protects against cerebral ischemia in the mouse. Stroke. 2009; 40(10):3349–55. doi: 10.1161/STROKEAHA.109.557314 19628803 10.1161/STROKEAHA.109.557314

[41] Karl T, Pabst R, von Hörsten S. Behavioral phenotyping of mice in pharmacological and toxicological research. Exp Toxicol Pathol. 2003; 55(1):69–83. doi: 10.1078/0940-2993-00301 12940631 10.1078/0940-2993-00301

[42] Boissier JR, Simon P, Giudicelli JF. Central effects of some adrenergic blocking and-or sympatholytic substances. II. Action on spontaneous motility and potentialization of pentobarbital. Arch Int Pharmacodyn Ther. 1967; 169(2):312–9. 6064561

[43] Barnes CA. Memory deficits associated with senescence: a neurophysiological and behavioral study in the rat. J Comp Physiol Psychol. 1979; 93(1):74–104. 221551 10.1037/h0077579

[44] File SE. Factors controlling measures of anxiety and responses to novelty in the mouse. Behav Brain Res. 2001; 125(1–2):151–7. 11682106 10.1016/s0166-4328(01)00292-3

[45] Smith C, Gentleman SM, Leclercq PD, Murray LS, Griffin WST, Graham DI, et al The neuroinflammatory response in humans after traumatic brain injury. Neuropathol Appl Neurobiol. 2013; 39(6):654–66. doi: 10.1111/nan.12008 23231074 10.1111/nan.12008 PMC3833642

[46] Kinnunen KM, Greenwood R, Powell JH, Leech R, Hawkins PC, Bonnelle V, et al White matter damage and cognitive impairment after traumatic brain injury. Brain J Neurol. 2011; 134(Pt 2):449–63. 10.1093/brain/awq347 PMC3030764 21193486

[47] Homsi S, Piaggio T, Croci N, Noble F, Plotkine M, Marchand-Leroux C, et al Blockade of acute microglial activation by minocycline promotes neuroprotection and reduces locomotor hyperactivity after closed head injury in mice: a twelve-week follow-up study. J Neurotrauma. 2010; 27(5):911–21. doi: 10.1089/neu.2009.1223 20166806 10.1089/neu.2009.1223

[48] Girgis H, Palmier B, Croci N, Soustrat M, Plotkine M, Marchand-Leroux C. Effects of selective and non-selective cyclooxygenase inhibition against neurological deficit and brain oedema following closed head injury in mice. Brain Res. 2013; 1491:78–87. doi: 10.1016/j.brainres.2012.10.049 23122881 10.1016/j.brainres.2012.10.049

[49] Morganti JM, Riparip L-K, Rosi S. Call Off the Dog(ma): M1/M2 Polarization Is Concurrent following Traumatic Brain Injury. PloS One. 2016;11(1):e0148001 doi: 10.1371/journal.pone.0148001 26808663 10.1371/journal.pone.0148001 PMC4726527

[50] Murray KN, Parry-Jones AR, Allan SM. Interleukin-1 and acute brain injury. Front Cell Neurosci. 2015;9:18 doi: 10.3389/fncel.2015.00018 25705177 10.3389/fncel.2015.00018 PMC4319479

[51] Shaftel SS, Griffin WST, O’Banion MK. The role of interleukin-1 in neuroinflammation and Alzheimer disease: an evolving perspective. J Neuroinflammation. 2008;5:7 doi: 10.1186/1742-2094-5-7 18302763 10.1186/1742-2094-5-7 PMC2335091

[52] Wu TC, Wilde EA, Bigler ED, Li X, Merkley TL, Yallampalli R, et al Longitudinal changes in the corpus callosum following pediatric traumatic brain injury. Dev Neurosci. 2010; 32(5–6):361–73 doi: 10.1159/000317058 20948181 10.1159/000317058 PMC3073757

[53] Hergenroeder GW, Redell JB, Moore AN, Dash PK. Biomarkers in the clinical diagnosis and management of traumatic brain injury. Mol Diagn Ther. 2008;12(6):345–58 19035622 10.1007/BF03256301

[54] Sandler SJI, Figaji AA, Adelson PD. Clinical applications of biomarkers in pediatric traumatic brain injury. Childs Nerv Syst. 2010; 26(2):205–13. doi: 10.1007/s00381-009-1009-1 19902222 10.1007/s00381-009-1009-1

[55] Liu MC, Akle V, Zheng W, Kitlen J, O’Steen B, Larner SF, et al Extensive degradation of myelin basic protein isoforms by calpain following traumatic brain injury. J Neurochem. 2006; 98(3):700–12. doi: 10.1111/j.1471-4159.2006.03882.x 16893416 10.1111/j.1471-4159.2006.03882.x

[56] Radtke C, Sasaki M, Lankford KL, Gallo V, Kocsis JD. CNPase expression in olfactory ensheathing cells. J Biomed Biotechnol. 2011; 2011:608496 doi: 10.1155/2011/608496 22174557 10.1155/2011/608496 PMC3228405

[57] Ajao DO, Pop V, Kamper JE, Adami A, Rudobeck E, Huang L, et al Traumatic brain injury in young rats leads to progressive behavioral deficits coincident with altered tissue properties in adulthood. J Neurotrauma. 2012; 29(11):2060–74. doi: 10.1089/neu.2011.1883 22697253 10.1089/neu.2011.1883 PMC3408248

[58] Ihara M, Polvikoski TM, Hall R, Slade JY, Perry RH, Oakley AE, et al Quantification of myelin loss in frontal lobe white matter in vascular dementia, Alzheimer’s disease, and dementia with Lewy bodies. Acta Neuropathol (Berl). 2010; 119(5):579–89. 20091409 10.1007/s00401-009-0635-8 PMC2849937

[59] Robinson AP, Harp CT, Noronha A, Miller SD. The experimental autoimmune encephalomyelitis (EAE) model of MS: utility for understanding disease pathophysiology and treatment. Handb Clin Neurol. 2014;122:173–89. doi: 10.1016/B978-0-444-52001-2.00008-X 24507518 10.1016/B978-0-444-52001-2.00008-X PMC3981554

[60] Scolding NJ, Houston WA, Morgan BP, Campbell AK, Compston DA. Reversible injury of cultured rat oligodendrocytes by complement. Immunology. 1989; 67(4):441–6. 2767708 PMC1385311

[61] Miller SD, Karpus WJ, Davidson TS. Experimental autoimmune encephalomyelitis in the mouse. Curr Protoc Immunol Ed John E Coligan Al. 2010; Chapter 15:Unit 15.1 doi: 10.1002/0471142735.im1501s88 20143314 10.1002/0471142735.im1501s88

[62] Donovan V, Kim C, Anugerah AK, Coats JS, Oyoyo U, Pardo AC, et al Repeated mild traumatic brain injury results in long-term white-matter disruption. J Cereb Blood Flow Metab. 2014; 34(4):715–23. doi: 10.1038/jcbfm.2014.6 24473478 10.1038/jcbfm.2014.6 PMC3982100

[63] Dams-O’Connor K, Spielman L, Singh A, Gordon WA, Lingsma HF, Maas AIR, et al The impact of previous traumatic brain injury on health and functioning: a TRACK-TBI study. J Neurotrauma. 2013; 30(24):2014–20. doi: 10.1089/neu.2013.3049 23924069 10.1089/neu.2013.3049 PMC3868372

[64] Donders J, Strong C-AH. Clinical utility of the Wechsler Adult Intelligence Scale-Fourth Edition after traumatic brain injury. Assessment. 2015; 22(1):17–22. doi: 10.1177/1073191114530776 24752385 10.1177/1073191114530776

[65] Yurgelun-Todd DA, Bueler CE, McGlade EC, Churchwell JC, Brenner LA, Lopez-Larson MP. Neuroimaging correlates of traumatic brain injury and suicidal behavior. J Head Trauma Rehabil. 2011; 26(4):276–89. doi: 10.1097/HTR.0b013e31822251dc 21734511 10.1097/HTR.0b013e31822251dc

[66] Washington PM, Forcelli PA, Wilkins T, Zapple DN, Parsadanian M, Burns MP. The Effect of Injury Severity on Behavior: A Phenotypic Study of Cognitive and Emotional Deficits after Mild, Moderate, and Severe Controlled Cortical Impact Injury in Mice. J Neurotrauma. 2012; 29(13):2283–96. doi: 10.1089/neu.2012.2456 22642287 10.1089/neu.2012.2456 PMC3430487

[67] Xuan W, Vatansever F, Huang L, Wu Q, Xuan Y, Dai T, et al Transcranial low-level laser therapy improves neurological performance in traumatic brain injury in mice: effect of treatment repetition regimen. PloS One. 2013; 8(1):e53454 doi: 10.1371/journal.pone.0053454 23308226 10.1371/journal.pone.0053454 PMC3538543

[68] Liu N-K, Zhang Y-P, Zou J, Verhovshek T, Chen C, Lu Q-B, et al A semicircular controlled cortical impact produces long-term motor and cognitive dysfunction that correlates well with damage to both the sensorimotor cortex and hippocampus. Brain Res. 2014;1576:18–26. doi: 10.1016/j.brainres.2014.05.042 24905625 10.1016/j.brainres.2014.05.042

[69] Szczygielski J, Mautes AE, Müller A, Sippl C, Glameanu C, Schwerdtfeger K, et al Decompressive Craniectomy Increases Brain Lesion Volume and Exacerbates Functional Impairment in Closed Head Injury in Mice. J Neurotrauma. 2016; 33(1):122–31. doi: 10.1089/neu.2014.3835 26102497 10.1089/neu.2014.3835

[70] O’Connor C, Heath DL, Cernak I, Nimmo AJ, Vink R. Effects of daily versus weekly testing and pre-training on the assessment of neurologic impairment following diffuse traumatic brain injury in rats. J Neurotrauma. 2003; 20(10):985–93. doi: 10.1089/089771503770195830 14588115 10.1089/089771503770195830

[71] Lima FD, Souza MA, Furian AF, Rambo LM, Ribeiro LR, Martignoni FV, et al Na+,K+-ATPase activity impairment after experimental traumatic brain injury: relationship to spatial learning deficits and oxidative stress. Behav Brain Res. 2008; 193(2):306–10. doi: 10.1016/j.bbr.2008.05.013 18573545 10.1016/j.bbr.2008.05.013

[72] Fedor M, Berman RF, Muizelaar JP, Lyeth BG. Hippocampal θ dysfunction after lateral fluid percussion injury. J Neurotrauma. 2010; 27(9):1605–15. doi: 10.1089/neu.2010.1370 20597686 10.1089/neu.2010.1370 PMC2966852

[73] Wang Y, Neumann M, Hansen K, Hong SM, Kim S, Noble-Haeusslein LJ, et al Fluoxetine increases hippocampal neurogenesis and induces epigenetic factors but does not improve functional recovery after traumatic brain injury. J Neurotrauma. 2011; 28(2):259–68. doi: 10.1089/neu.2010.1648 21175261 10.1089/neu.2010.1648 PMC5206695

[74] Zhao Z, Loane DJ, Murray MG, Stoica BA, Faden AI. Comparing the predictive value of multiple cognitive, affective, and motor tasks after rodent traumatic brain injury. J Neurotrauma. 2012; 29(15):2475–89. doi: 10.1089/neu.2012.2511 22924665 10.1089/neu.2012.2511 PMC3471125

[75] Chou A, Morganti JM, Rosi S. Frontal Lobe Contusion in Mice Chronically Impairs Prefrontal-Dependent Behavior. PloS One. 2016; 11(3):e0151418 doi: 10.1371/journal.pone.0151418 26964036 10.1371/journal.pone.0151418 PMC4786257

[76] Bondi CO, Cheng JP, Tennant HM, Monaco CM, Kline AE. Old dog, new tricks: the attentional set-shifting test as a novel cognitive behavioral task after controlled cortical impact injury. J Neurotrauma. 2014; 31(10):926–37. doi: 10.1089/neu.2013.3295 24397572 10.1089/neu.2013.3295 PMC4012626

[77] Leunissen I, Coxon JP, Caeyenberghs K, Michiels K, Sunaert S, Swinnen SP. Subcortical volume analysis in traumatic brain injury: the importance of the fronto-striato-thalamic circuit in task switching. Cortex. 2014; 51:67–81. doi: 10.1016/j.cortex.2013.10.009 24290948 10.1016/j.cortex.2013.10.009

