# Supplementary figures and images for: Neuroinflammation, myelin and behavior: Temporal patterns following mild traumatic brain injury in mice
Source: PLoS One. 2017 Sep 14;12(9):e0184811. doi: 10.1371/journal.pone.0184811 (PMC5599047; doi:10.1371/journal.pone.0184811)

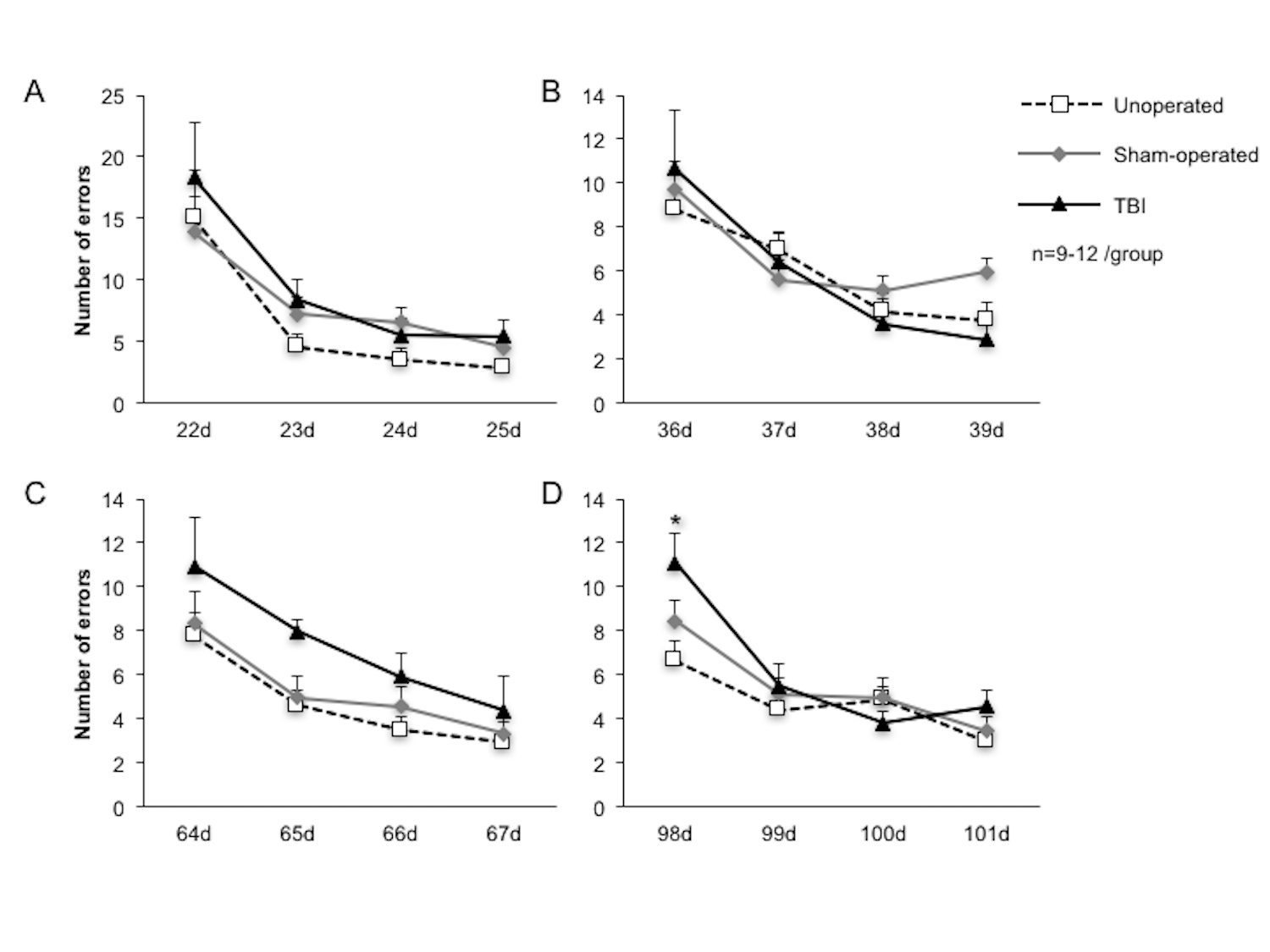

Supplement: S1 Fig — The number of errors was obtained during the Barnes maze test, performed on unoperated, sham-operated and TBI mice at (A) 15 days, (B) 1 month, (C) 2 months and (D) 3 months post-injury. Data were expressed as means ± S.E.M. Differences were analyzed by 2-way ANOVA for repeated measures, followed by a Dunnett test with Bonferroni correction. *p < 0.05 vs sham-operated. (TIF) [file pone.0184811.s001.tif]

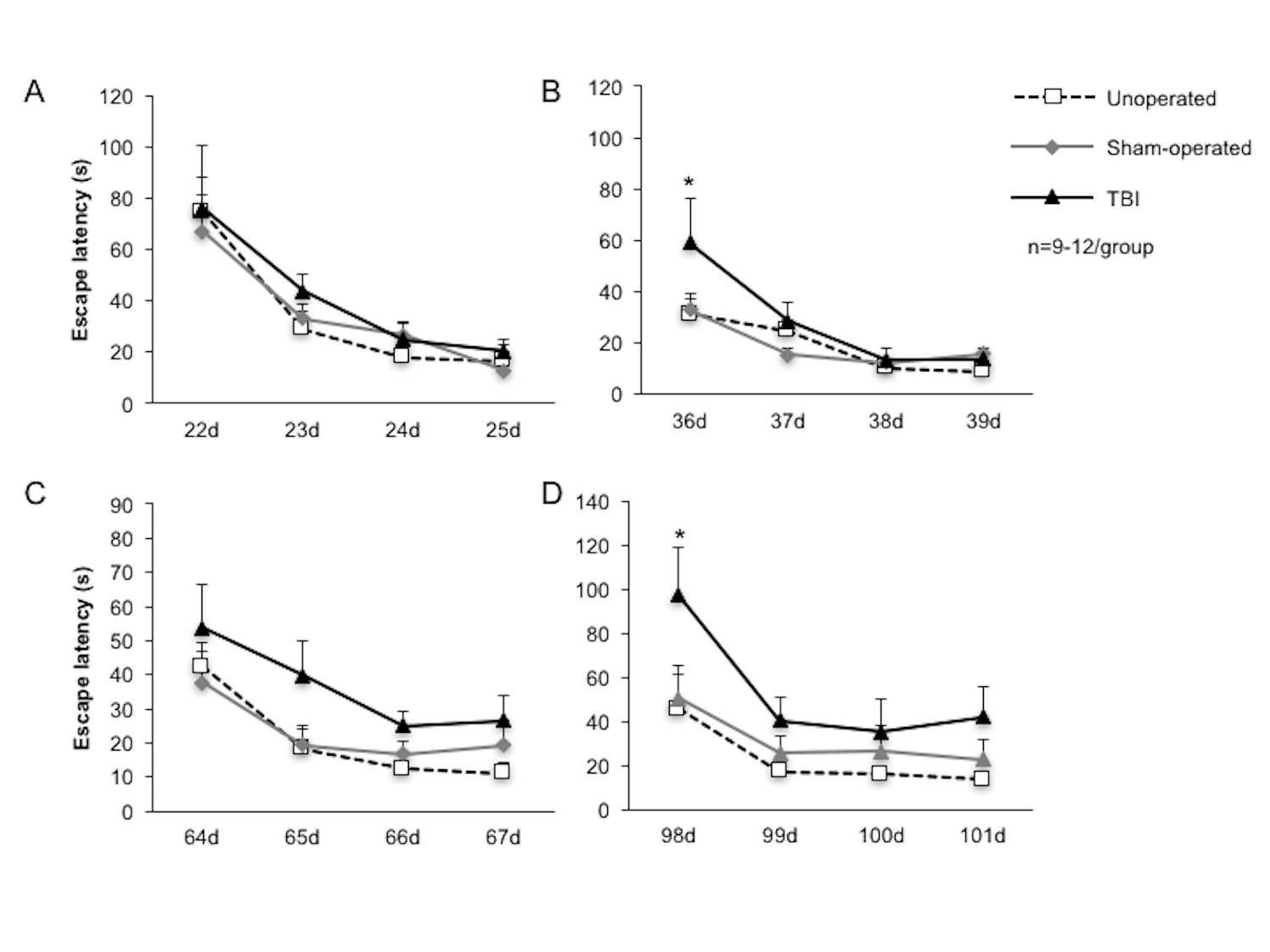

Supplement: S2 Fig — The escape latency was obtained during the Barnes maze test, performed on unoperated, sham-operated and TBI mice at (A) 15 days, (B) 1 month, (C) 2 months and (D) 3 months post-injury. Data were expressed as means ± S.E.M. Differences were analyzed by 2-way ANOVA for repeated measures, followed by a Dunnett test with Bonferroni correction. *p < 0.05 vs sham-operated. (TIF) [file pone.0184811.s002.tif]
